# Ferroelectricity through Reversible Anion-Relay Polarization Switching in a Two-Dimensional Metal–Organic Framework

**DOI:** 10.1021/jacs.5c18104

**Published:** 2026-02-19

**Authors:** Neetu Prajesh, Vikash Kushwaha, Chandan K. Singh, Vijay Bhan Sharma, Balu Praveenkumar, Alexander Steiner, Maciej Ptak, Dinesh Kabra, Jan K. Zaręba, Ramamoorthy Boomishankar

**Affiliations:** † Department of Chemistry, 193158 Indian Institute of Science Education and Research (IISER), Pune, Dr. Homi Bhabha Road, Pune 411008, India; ‡ Department of Physics, 193158 Indian Institute of Science Education and Research (IISER), Pune, Dr. Homi Bhabha Road, Pune 411008, India; § Department of Physics, 29491 Indian Institute of Technology, Bombay, Powai, Mumbai 400076, India; ∥ Armament Materials & Technology Centre, Armament Research and Development Establishment (ARDE), Defence Research and Development Organisation (DRDO), Dr. Homi Bhabha Road, Pune 411021, India; ⊥ Department of Chemistry, University of Liverpool, Crown Street, Liverpool L69 7ZD, United Kingdom; # 215275 Institute of Low Temperature and Structure Research, Polish Academy of Sciences, Okólna 2, Wrocław 50-422, Poland; ∇ Institute of Advanced Materials, Faculty of Chemistry, Wroclaw University of Science and Technology, 50-370 Wroclaw, Poland

## Abstract

Ferroelectric materials are central to next-generation electronics and energy technologies because of their ability to couple electrical, mechanical, and thermal signals. Metal–organic frameworks (MOFs) provide a versatile platform for such functionalities owing to their structural tunability; however, despite notable examples, the microscopic mechanisms governing polarization switching in MOFs remain poorly understood. Here we report a Cu­(II)-based polar two-dimensional metal–organic framework [Cu­(PhPO­(NHCH_2_^3^Py)_2_)]­(NO_3_)_2_·2H_2_O (**1·2H**_**2**_ **O**), constructed from a low-symmetric flexible dipodal phosphoramide ligand, PhPO­(NHCH_2_^3^Py)_2_. Compound **1·2H**_**2**_ **O** exhibits robust ferroelectricity, confirmed by a well-defined rectangular *P*–*E* hysteresis loop with a saturation polarization of 1.2 μC/cm^2^. The ferroelectric polar domains, along with bias-dependent amplitude-butterfly and phase-hysteresis loops, were characterized by piezoresponse force microscopy (PFM). First-principles calculations uncover an unusual displacive polarization-switching pathway, in which two nitrate ions displace together along a field-defined direction, enabling reversible 180° dipole reversal through bonding reorganization at the Cu­(II) center. This reversible anion-relay mechanism expands the catalog of microscopic ferroelectric processes and represents a new paradigm for MOFs. To demonstrate practical utility, flexible piezoelectric nanogenerators (PENGs) were fabricated by embedding **1·2H**_**2**_ **O** in thermoplastic polyurethane composites. The champion 10 wt % device delivered an open-circuit voltage of 25.1 V and a maximum power density of 48.7 μW/cm^2^, highlighting the potential of MOF-based ferroelectrics for piezoelectric energy harvesting applications.

## Graphical abstract

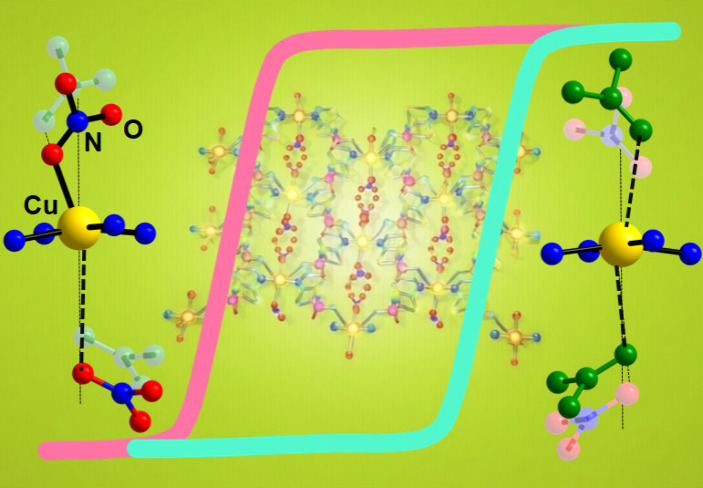

## Introduction

Polar materials with switchable functionalities exhibit the capacity to respond to external stimuli through changes in their polarization state. Such switchable polarizations are often associated with either subtle or pronounced structural distortions, which, in turn, give rise to a variety of coupled physical phenomena. The interaction between polar order and lattice dynamics underpins functional properties such as piezoelectricity, pyroelectricity, and ferroelectricity, enabling the transduction of mechanical, thermal, or electrical inputs into measurable outputs.^1–3^ Among these, ferroelectricity refers to the spontaneous occurrence of electric polarization that can be reversibly switched between symmetry-equivalent states under an applied electric field. This phenomenon serves as the basis for applications in nonvolatile memories, mechanical switches, sensors, actuators, and energy-harvesting devices.

Polarization switching in ferroelectrics arises from diverse microscopic mechanisms. In classical oxide ferroelectrics such as BaTiO_3_ and PbTiO_3_, switching involves displacive transitions, in which metal cations shift off-center within a noncentrosymmetric lattice.^4,5^ In molecular systems including NaNO_2_ and diisopropylammonium salts,^6,7^ polarization emerges from order–disorder transitions, as thermally disordered dipoles align upon cooling. The hydrogen-bonded ferroelectrics, such as KH_2_PO_4_ and DABCO-based salts, combine proton-transfer and ionic displacement along O–H···O hydrogen bonds, thus integrating both order–disorder and displacive mechanisms.^8^ Molecular ferroelectrics can display even more complex switching processes, sometimes involving molecular reorganization or modification of coordination bonds, as observed in [(CH_3_)_3_NOH]_3_[KFe­(CN)_6_] and related salts.^9–11^ Nevertheless, in most cases, the primary switching mechanism typically traces back to order–disorder transitions of dipolar or guest species. More recently, additional mechanisms such as interlayer sliding, bowl–to–bowl inversion, and electron transfer have also been reported.^12–14^ Metal–organic frameworks (MOFs), a prominent subclass of molecular ferroelectrics, embody many of these mechanisms, owing to their flexible coordination networks and polar components.

In reported MOF-based ferroelectrics, polarization typically originates from the rotation of dipolar substituents on organic linkers,^15,16^ the substitution of metal ions at lattice nodes,^17^ or the ordering of counteranions and polar guest molecules.^18–22^ However, despite these advances, the fundamental mechanisms governing polarization switching in MOFs remain incompletely understood. In contrast to classical inorganic ferroelectrics, many MOFs exhibit dynamic structural flexibility without distinct phase transitions, which complicates the identification of their polarization-switching pathways. Progress in this field thus requires the discovery of MOF systems with clearly defined ferroelectric origins and the elucidation of their molecular-level switching behavior.

MOFs also offer an attractive platform for chemical functionalization, providing functionalities not readily available in solid-state oxides. Yet, most MOFs crystallize in centrosymmetric space groups due to the symmetric nature of their metal–oxo secondary building units (SBUs) and the ordered topology of their frameworks. To address this limitation, we have designed lower-symmetric di- and tripodal (N-donor or O-donor) phosphoramide ligands, including [PhPO­(NHPy)_2_], (Py = 3-pyridyl (^3^Py) or 4-pyridyl (^4^Py)), [PS­(NH^3^Py)_3_], [PO­(NHCH_2_^3^Py)_3_] and [PhPO­(NH-(C_6_H_4_COOH))_2_]. These ligands enable the formation of coordination networks with ferro- and piezoelectric properties. Using this approach, we have developed noncentrosymmetric metal–organic cages and their connected cage frameworks, as well as one- and two-dimensional polar coordination networks, in which polarization originates from either charge-separated structural motifs or distortions around the metal centers.^23–31^

Herein, we present a polar 2D metal–organic framework, [Cu­(PhPO­(NHCH_2_^3^Py)_2_)]­(NO_3_)_2_·2H_2_O (**1·2H**_**2**_ **O**), synthesized using the dipodal phosphoramide ligand [PhPO­(NHCH_2_^3^Py)_2_] (**L**). Structural and computational analyses reveal a previously unrecognized microscopic phenomenon in metal–organic systems: an electric-field-driven, axially coordinated anion-relay process that functions as a distinct ferroelectric switching mechanism. In this case, reversible displacements of two axially bound nitrate ions modulate their interactions with the Cu­(II) center under an applied electric field. The ferroelectric nature of **1·2H**_**2**_ **O** was confirmed by a well-defined rectangular *P*–*E* hysteresis loop with a saturation polarization (*P*_s_) of 1.2 μC/cm^2^. Furthermore, piezoresponse force microscopy (PFM) measurements revealed charecteristic butterfly and phase hysteresis loops under an applied bias, consistent with ferroelectric and piezoelectric behavior of **1·2H**_**2**_ **O**. Complementing these nanoscale observations, the macroscopic piezoelectric coefficient (*d*_33_) measured via the quasi-static Berlincourt method on a pelletized, poled sample of **1·2H**_**2**_ **O** was found to be 29.4 pC/N. To harness these properties for practical applications, composite piezoelectric nanogenerators (PENGs) were fabricated by embedding **1·2H**_**2**_ **O** in a thermoplastic polyurethane (TPU) matrix. The optimized composite device (10 wt % loading) yielded an output voltage of 25.1 V and a power density of 48.7 μW/cm^2^. Overall, these findings introduce a new “tug-of-war” like mechanism for ferroelectric switching in coordination frameworks and demonstrate how embedding such MOFs into flexible composites can translate intrinsic properties into functional devices, paving the way for future energy and sensing applications.

## Results and Discussion

### Synthesis, Characterization, and Crystal Structures

The flexible dipodal ligand **L** was synthesized by refluxing 3-picolylamine and PhPOCl_2_ in toluene (Scheme S1). The ligand was characterized by using NMR spectroscopy and single-crystal X-ray diffraction analysis (Figures S1–S4). The two-dimensional metal–organic framework **1** was obtained as blue crystals from a 2:1 reaction mixture of **L** and Cu­(NO_3_)_2_·3H_2_O in a MeOH/H_2_O system at room temperature (Scheme 1). The structural and thermogravimetric analyses indicated that the as-synthesized crystals have a layered 2D coordination framework of the composition CuL_2_(NO_3_)_2_· 3H_2_O (**1·3H**_**2**_ **O**) (**L** = PhPO­(NHCH_2_^3^Py)_2_). Storing these crystals at room temperature for a prolonged period (>24 h) in a desiccator, or sonicating them in a hexane solution at 40 °C, resulted in the loss of one water molecule per formula unit, yielding **1·2H**_**2**_ **O** as the stable phase (vide supra).

**Scheme 1:**
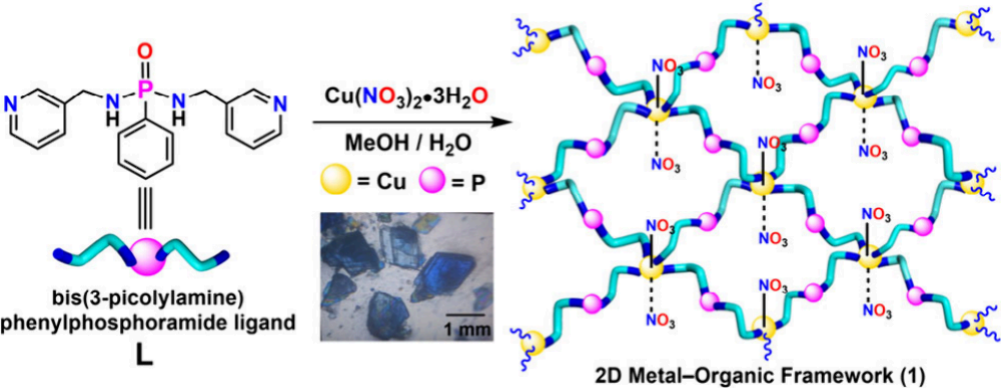
Schematic Diagram Showing the Formation of Two-Dimensional Metal–Organic Framework **1** from the Dipodal Picolyl-Based Phosphoramide Ligand **L** ^a^

a Images of the crystals of **1·3H**_**2**_ **O** under the microscope showing the layered morphology.

Single-crystal X-ray diffraction (SCXRD) of a crystal of the compound, taken fresh from the mother liquor, was carried out at 150 K. The structure of **1·3H**_**2**_ **O** was solved in orthorhombic space group symmetry *Aea*2 (Figure 1a and Figure S5). The dipodal phosphoramide ligand connects two Cu­(II) ions via its pyridyl groups. The Cu­(II) ion resides on a crystallographic 2-fold axis and is coordinated by four pyridyl groups and one nitrate ion (N30 set) in a square-pyramidal fashion with the nitrate ion in the apical position. The Cu–N bonds to the ligands are shorter (2.024(6) and 2.026(6) Å) than the Cu–O bond to the coordinated nitrate ion (Cu–O31: 2.313(10) Å); this elongation can be attributed to the Jahn–Teller effect characteristic of the d^9^ metal ion.^32^ This coordination environment gives rise to a 2D layered framework with an ABAB stacking arrangement in which the intralayer voids are filled by the uncoordinated nitrate anion (N40 set) and the three water molecules (Figures 1b, S6 and S7).

**Figure 1:**
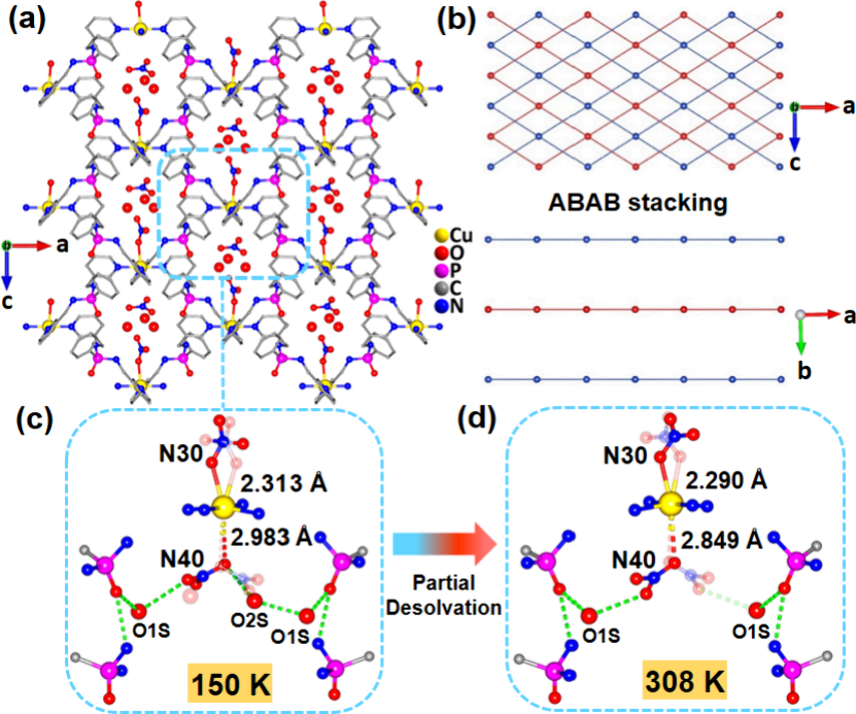
(a) Crystal structure of **1·3H**_**2**_ **O** at 150 K. (b) Stacking view of the cationic layers in both **1·3H**_**2**_ **O** and **1·2H**_**2**_ **O** along the *b*- and *c*-axes. The zoomed-in view of the coordinating sites at the Cu­(II) centers in (c) **1·3H**_**2**_ **O** at 150 K and (d) **1·2H**_**2**_ **O** at 308 K.

This nitrate ion is close to the open coordination site of the metal center but too far from it to count as a genuine coordination (Cu–O41: 2.98 Å). Both coordinated (centered on N30) and noncoordinated (centered on N40) nitrate ions are disordered over the 2-fold axis running through the Cu­(II) ions. One water molecule (O2S) is involved in the disorder, swapping its position with the uncoordinated nitrate ion. The two remaining water molecules (O1S) are equivalent by symmetry and reside at opposite ends of the cavity, where they are hydrogen-bonded to phosphoramide units (Figure 1c). In addition, these O1S atoms were found to interact with the nitrate ion from the N40 set and the other water molecule (O2S).

Partial dehydration occurs when the crystal is warmed to room temperature. SCXRD analysis performed at 308 K indicates the loss of one water molecule (O2S) per formula unit (the site previously involved in disorder with the uncoordinated nitrate ion), whereas the two more tightly bound water molecules (O1S) remain in the lattice. This yield an overall composition CuL_2_(NO_3_)_2_·2H_2_O (**1·2H**_**2**_ **O**) (Figure S8 and S9). The remainder of the structure exhibits minimal alteration, with the exception that both of the O1S atoms interact only with the uncoordinated nitrate anions within the cavity. Notably, the Cu–O41 distance to the uncoordinated nitrate ion is shortened to 2.85 Å, with a change in distance of 0.13 Å (Figure 1d). This suggests that the nitrate ions can undergo displacement along the *c*-axis in addition to the disorder observed along the *b*-axis.

### Temperature-Dependent Raman, TGA, DSC, and SHG Studies

To better understand the dynamics of the nitrate ions in **1·2H**_**2**_ **O**, temperature-dependent Raman spectra were collected (Figure 2a). At 80 K, most bands are narrow, indicating that the dynamics of ligands and nitrate ions are suppressed. Upon heating to 350 K, all Raman bands exhibit continuous broadening, softening, and a decrease in intensity, consistent with the typical thermal behavior of phonon modes. The widening of adjacent bands and their merging into broader contours, likely due to thermal effects, confirm that the orthorhombic space-group symmetry of *Aea*2 remains unchanged over the studied temperature range. Special attention was focused on the narrow spectral range of 1010–1080 cm^–1^, where a strong contribution from the symmetric stretching mode, ν_s_, is expected (Figure 2b).^33^ Analysis of the remaining three bands associated with other internal modes of the nitrate ions was not possible due to their low intensity even at 80 K (Figure 2a). Both ν_s_ bands, observed at 1037 and 1052 cm^–1^, soften as the temperature rises (Figure 2c); however, their thermal behavior differs, confirming the presence of two distinct nitrate ions with varying environments in the crystal structure of **1·2H**_**2**_ **O**. The band at 1052 cm^–1^ softens more rapidly, supporting its assignment to an uncoordinating nitrate ion with greater freedom, which allows it to approach the central Cu­(II) ion. The second Raman band at 1037 cm^–1^ is attributed to the nitrate ion within the octahedral coordination sphere of the metal ion; therefore, it has a restricted mobility. The greater molecular liberation of the more distant nitrate ion is also evident from the clearly stronger broadening of the 1052 cm^–1^ band during the heating event (Figure 2b).

**Figure 2:**
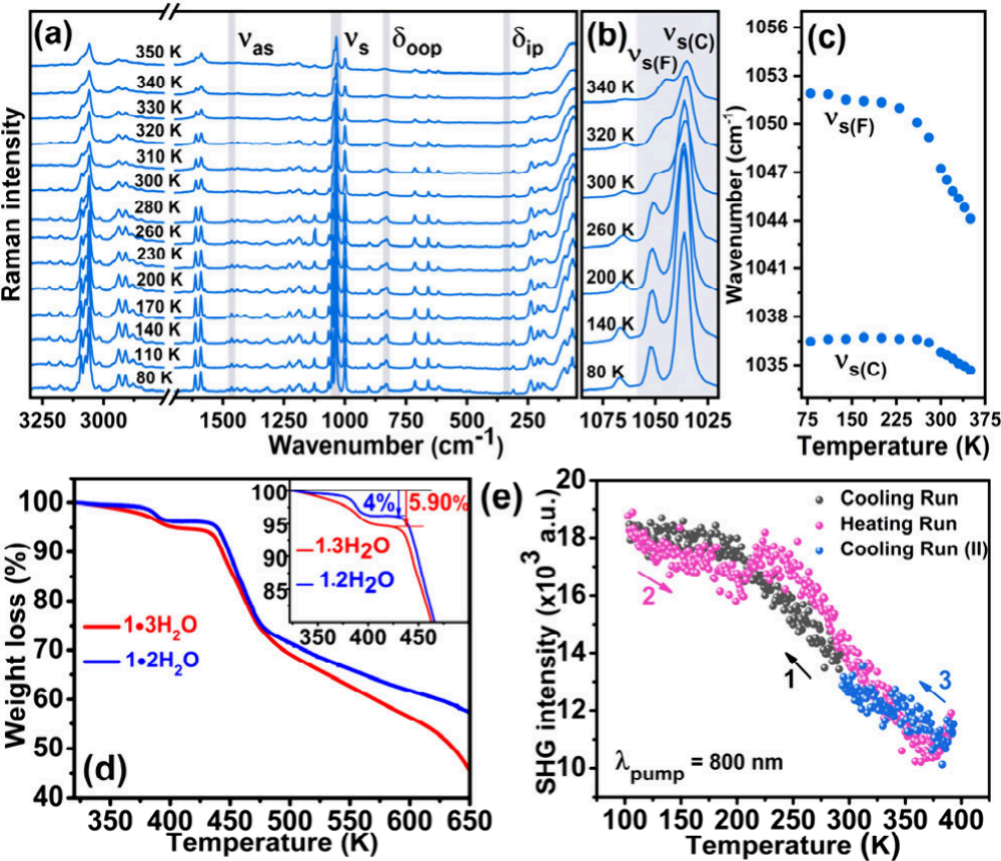
(a) Thermal evolution of Raman spectra of **1·2H**_**2**_ **O**; ν_as/s_ denotes antisymmetric/symmetric stretching, and δ_ip/oop_ denotes the in-plane/out-of-plane bending of the nitrate ion. Thermal evolution of Raman spectra (b) in the range corresponding to symmetric modes of the nitrate ions (ν_s_), and (c) changes in band positions; C and F subscripts refer to coordinated and free nitrate ions, respectively. (d) Thermogravimetric data of **1·3H**_**2**_ **O** and **1·2H**_**2**_ **O** (inset: closer view of the desolvation profiles). (e) Second harmonic generation vs temperature plot of **1·2H**_**2**_ **O**.

Thermogravimetric analysis (TGA) of fully solvated (**1·3H**_**2**_ **O**) and partially desolvated (**1·2H**_**2**_ **O**) samples reveals clear signatures in the weight-loss profiles (Figures 2d and S11a). For **1·3H**_**2**_ **O** (red curve), the TGA profile shows a continuous weight loss from 322 K, followed by an abrupt weight loss from 375 to 433 K. The initial weight loss of 1.96% observed up to 358 K corresponds to the release of one lattice H_2_O, while the overall weight loss of 5.90%, from 322 to 433 K, is consistent with the loss of all three water molecules. For **1·2H**_**2**_ **O** (blue curve), a sharp mass loss is observed starting around 375 K and ending near 433 K. This corresponds to a weight loss of 4.0%, matching the release of two water molecules, in alignment with the crystal structure determined at 308 K. Nevertheless, both **1·3H**_**2**_ **O** and **1·2H**_**2**_ **O** exhibited stability up to 433 K, indicating the robustness of the CuL_2_(NO_3_)_2_ 2D framework.

The water molecule of the dihydrate (in **1·2H**_**2**_ **O**) is an integral part of the supramolecular structure. These water molecules act as a bridge between adjacent ligand units via hydrogen bonding to phosphamide groups: one via an OH···OP interaction and the other via an O···HN interaction, while also forming an interaction with one of the nitrate ions (Figures S9 and S10). These strong interactions rigidly reinforce the structural framework, requiring significantly higher thermal energy to disrupt the lattice. On the other hand, the extra water molecule in the trihydrate does not form a direct interaction with the framework but sits more loosely inside the cavity occupied by the disordered nitrate ions. Additionally, the water adsorption isotherms measured on an activated sample of **1·2H**_**2**_ **O** show only surface adsorption, with no uptake of bulk water (Figure S12). Differential scanning calorimetry (DSC) measurements show no detectable peak between 130 and 350 K. This is consistent with the crystallographic data, which indicates that both the low- and high-temperature structures retain orthorhombic *Aea*2 symmetry, without discontinuous structural change (Figure S11b).

To further investigate phase stability, variable-temperature single-crystal X-ray diffraction (VT–SCXRD) measurements were carried out between 150 and 353 K for **1·3H**_**2**_ **O**. The measurements indicate a small increase in all three axes (Figures S13 and S14). Notably, no clear Curie point corresponding to a ferroelectric–paraelectric transition was observed up to 353 K, reflecting the framework’s structural robustness. In fact, it should be noted that cooling the crystal back to 298 and 150 K after annealing it at 340 K for 1 h showed no reabsorption of the desolvated water molecule, confirming **1·2H**_**2**_ **O** as the stable phase as determined from their crystal structure analysis. A comparison of the unit cell volumes of **1·3H**_**2**_ **O** and **1·2H**_**2**_ **O** at 150 K shows a reduction of 185 Å^3^ (Table S3). This is consistent with the loss of one water molecule per formula unit, considering its molecular volume of 40 Å^3^ in a confined packing environment.^34^ Variable-temperature second harmonic generation (VT–SHG) measurements on the polycrystalline sample of **1·2H**_**2**_ **O** in the range of 100 to 383 K complement these findings, showing an SHG signal at low temperature that gradually decreases with increasing temperature, attributed to enhanced disorder of the nitrate anions at higher temperatures. Notably, no marked change in SHG intensity was observed within this temperature range, confirming the absence of any phase transitions (Figure 2e and Figure S15).

### Dielectric, Ferroelectric, and Piezoresponse Force Microscopy (PFM) Studies

Building on these structural and thermal insights, temperature-dependent dielectric measurements were performed on a silver-pasted pelletized sample of **1·2H**_**2**_ **O** (thickness: ∼0.5 mm; frequency: 1 kHz–100 kHz; *V*_ac_ = 1 V) to probe the dipolar dynamics as a function of temperature. The real part of the dielectric permittivity (*ε*′) exhibits a slightly broad feature between 330 and 378 K, signaling the dipolar fluctuations caused by the movement of the solvated water molecules and the enhancements in the nitrate disorder.^35–37^ A sharp increase in the *ε*′ values above 380 K is attributed to the onset of desolvation coupled with the increased mobility of the ions in the framework at higher temperatures (Figure 3a). This trend is also reflected in the dielectric loss (tan δ) data (Figure S16). In addition, the frequency-dependent dielectric profiles (*ε*′ and tan δ) follow trends similar to those of the temperature-dependent results (Figures S17 and S18). At room temperature, however, **1·2H**_**2**_ **O** displays a dielectric constant of ca. 23.0 and a low dissipation factor (tan δ), indicating good dielectric behavior and suggesting potential for practical applications.

**Figure 3:**
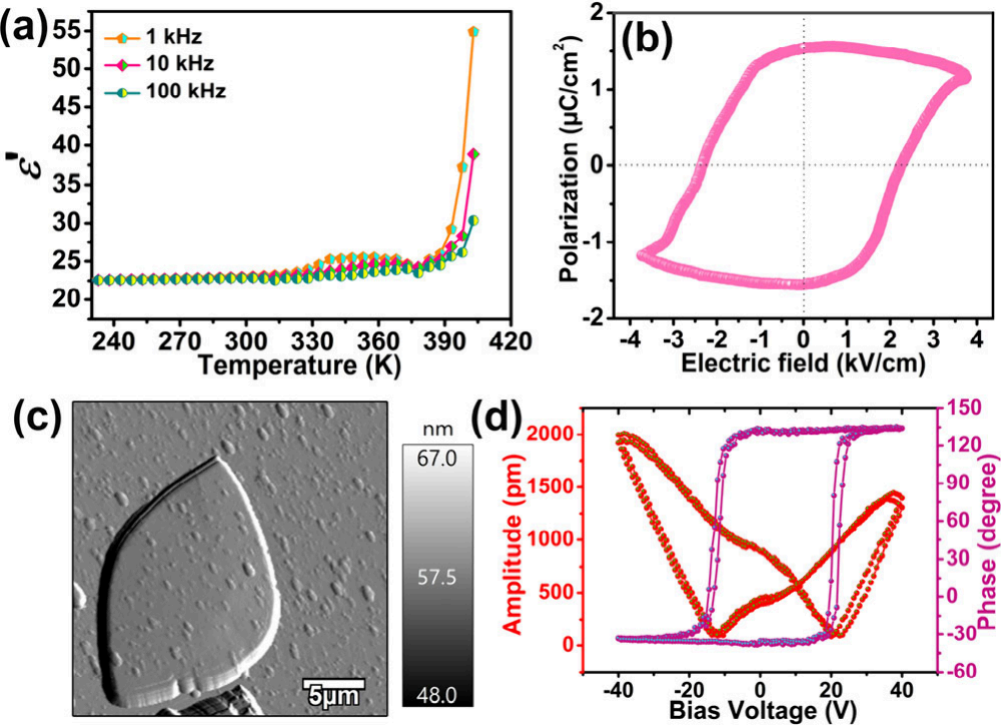
(a) Real part of dielectric permittivity *ε*′ vs temperature plot of **1·2H**_**2**_ **O**. (b) The *P*–*E* hysteresis loop of **1·2H**_**2**_ **O**. Vertical PFM data of **1·2H**_**2**_ **O**: (c) topography of single crystalline thin film of **1·2H**_**2**_ **O**, (d) vertical PFM amplitude and phase responses as a function of DC bias for a selected point of **1·2H**_**2**_ **O**, displaying local amplitude butterfly and phase hysteresis loops with a coercive voltage of nearly 20 V.

To further establish its ferroelectric behavior, polarization–electric field (*P*–*E*) hysteresis loop measurements were carried out at room temperature using a Sawyer–Tower circuit together with a Positive-Up-Negative-Down (PUND) protocol. For this purpose, a drop-cast polycrystalline film (thickness: ∼80 μm) of **1·2H**_**2**_ **O** was prepared on an indium tin oxide (ITO)-coated glass substrate (Figure S19). The measurements performed at room temperature yielded a well-defined rectangular hysteresis loop with a saturation polarization (*P*_s_) of 1.2 μC/cm^2^ and a coercive field (*E*_c_) of 2.2 kV/cm at 20 Hz, confirming reliable ferroelectric switching in **1·2H**_**2**_ **O**. The experimentally measured polarization is lower than the theoretical value calculated from the point charge model because the thin film is polycrystalline, whereas the calculation assumes an ideal single crystal (Figure S20). In a polycrystalline film, the polarization vectors of individual grains are not perfectly aligned with the film normal, resulting in a reduced effective polarization.^38^ Ferroelectric fatigue measurements of up to 10^6^ cycles demonstrated retention of the ferroelectric behavior (Figure S21).

Piezoresponse force microscopy (PFM), a nondestructive technique based on the local converse piezoelectric effect, is widely used to characterize piezoelectric and ferroelectric materials through a combination of surface imaging and spectroscopy modes.^39,40^ PFM images primarily consist of amplitude and phase signals, which correspond to the strength of the piezoelectric response and the polarization direction within individual domains, respectively.^41^ Vector-mode vertical PFM (VPFM) measurements were performed at room temperature on the as-grown, drop-casted single-crystalline thin film of **1·2H**_**2**_ **O**, which displayed good crystallinity as verified by PXRD (Figure S19). The probed region contained only a few micrometer-sized single crystals. The topography image (Figure 3c) reveals a layered morphology, while the corresponding PFM amplitude and phase images (Figures S22a and S22b) display well-defined stripe-like ferroelectric domain patterns. The contrast observed in the amplitude image, without crosstalk from topography, further supports the presence of intrinsic ferroelectric domains in the thin film. Measurements performed on another region of the film also exhibit consistent polar domain patterns, confirming robust, reproducible ferroelectric behavior across the sample (Figure S23).

PFM spectroscopy was further carried out at a selected point by applying a DC voltage of ± 40 V, superimposed with an AC signal of 2.5 V, to obtain off-field hysteresis loops. The off-field loops primarily reflect the electromechanical response arising from piezoelectricity, effectively eliminating contributions from electrostatic artifacts typically present in on-field mode.^42^ As shown in Figure 3d, the off-field hysteresis loops confirm the intrinsic ferroelectric nature of **1·2H**_**2**_ **O**. The rectangular, well-defined phase-hysteresis loop, exhibiting successive 180° polarization reversals, demonstrates the robust ferroelectricity of the thin film. The relatively modest coercive voltage of nearly 20 V, consistent with macroscopic *P*–*E* measurements and local PFM loops, indicates that the single-crystalline thin film exhibits highly responsive and efficiently switchable ferroelectric behavior. Additionally, the clean, characteristic butterfly shaped amplitude loop confirms the presence of piezoelectricity, which is challenging to achieve in thin-film MOFs.^15,18,27,30,43^ Furthermore, the macroscopic piezoelectric coefficient (*d*_33_) measured via the quasi-static Berlincourt method on a pelletized poled sample of **1·2H**_**2**_ **O** was determined to be 29.4 pC/N, demonstrating a good piezoelectric performance for MOF-based materials (Figure S24).

### Microscopic Origin of Ferroelectric Switching

In the crystal structure, positive charges are primarily localized on the Cu­(II) ions, while negative charges reside on the NO_3_^–^ ions. Room-temperature SCXRD data indicate that **1·2H**_**2**_ **O** crystallizes in the orthorhombic *Aea*2 space group, where the polarization axis corresponds to the crystallographic *c*-axis, along which the axially coordinated nitrate anions are aligned. It should be noted that the Cu­(II) phosphoramide framework adopts a rigid polar architecture (characterized by unidirectional P=O bonds; see Figure S25) that precludes inversion by an external electric field. Rather, ferroelectric switching becomes possible via the movement of nitrate ions toward or away from the Cu­(II) ions and subsequently between their disordered positions across the *c*-axis. In fact, the preferential switching of the nitrate anions across the *c*-axis to a specific disordered site breaks the symmetry of the crystal, reducing it from *Aea*2 to *Ab*11 (*P*c).

To investigate how the movement of nitrate ions along the polar *c*-axis affects the polarization, differential charge density analyses were performed by comparing charge distributions with and without an applied electric field along the *c*-axis. These calculations reveal a distinctive displacive-type mechanism: the axially aligned, weakly coordinated nitrate ions respond dynamically to the applied field, giving rise to reversible local dipoles. Under a moderate field of 0.1 eV/Å applied along the −*z* direction, the nitrate ions shift asymmetrically, changing polarization along the polar axis. Reversing the field induces an opposite distortion. Figures 4a and b shows a reversal of the sign of the differential charge density around the Cu atom upon reversal of the electric field direction. In addition to this metal-centered response, the applied field also induces electronic polarization of the ligand atoms (Figure S26). The induced dipole moments, computed from the differential charge density, are approximately 107 D per unit cell for an electric field applied along the −*z* direction and 72 D per unit cell for the +*z* direction. The robustness of this response is maintained up to a field of 1 eV/Å. Partial density of states (PDOS) analysis highlights key contributions from Cu, N, and O states near the Fermi level, validating their role in field-induced polarization (Figure S27). This field-tunable motion of axially coordinated anions represents a distinct ferroelectric switching mechanism in MOFs, driven by subtle, directional displacements that are mediated by metal-ion interaction rather than conventional lattice or ligand distortions.

**Figure 4:**
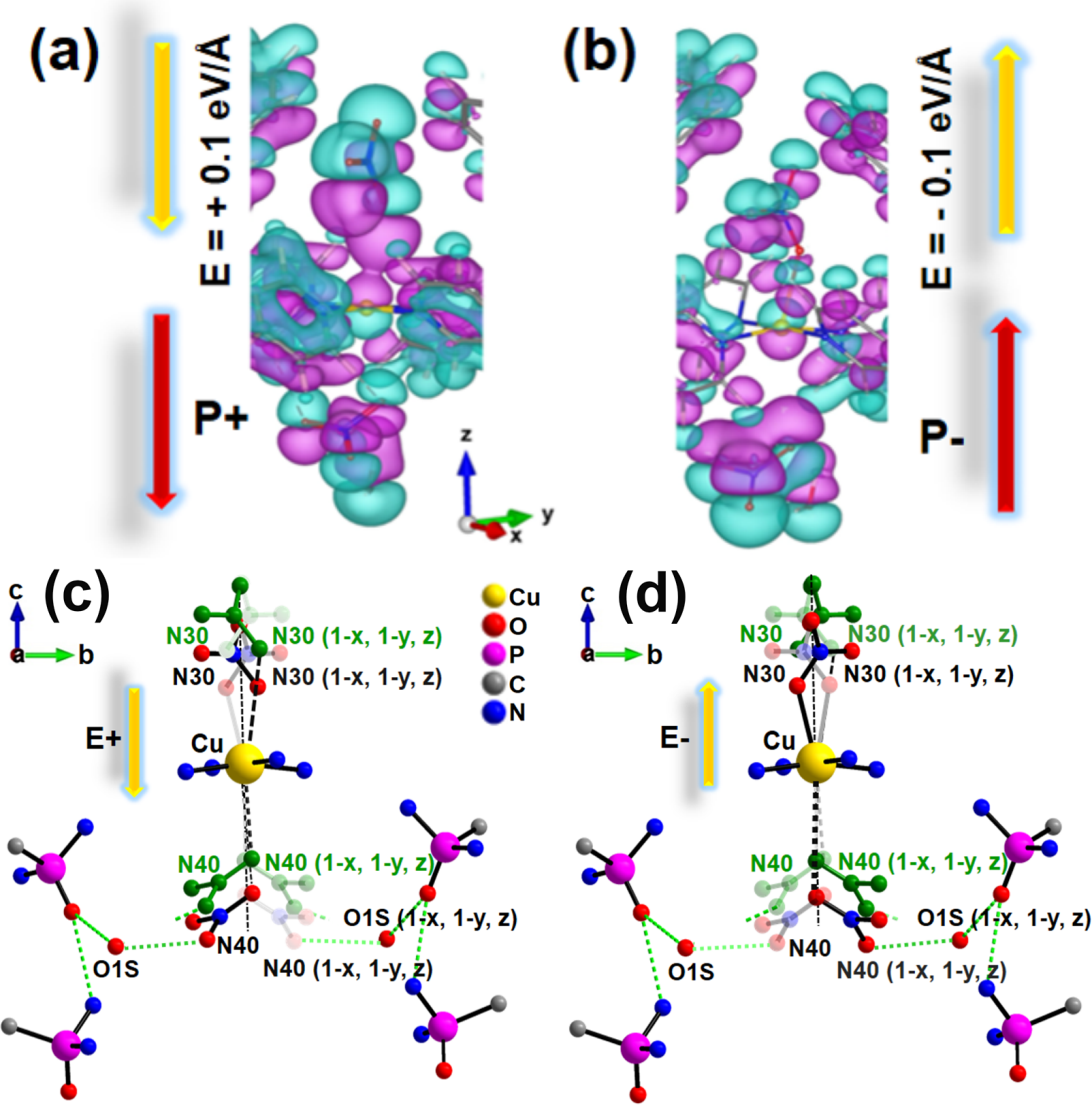
(a and b) Differential charge density plots under an external electric field along the *z*-direction for the optimized structure of **1·2H**_**2**_ **O**. (a) At 0.1 eV/Å, one NO_3_^–^ ion moves closer to the metal ion, while the other moves away, both along +*z*. (b) Upon field reversal, both NO_3_^–^ ions move along −*z*, with an inverted charge distribution relative to those depicted in Figure (a). Aqua and purple regions indicate charge accumulation and depletion, respectively. (c, d) Schematic illustration showing the proposed displacement of nitrate anions in **1·2H**_**2**_ **O** along the *c*-axis under the applied positive and negative electric fields, respectively. The nitrate positions indicated in green show the apparent movement of these ions under the electric field.

Further, potential evidence for the movement of nitrate ions can be observed from the X-ray photoelectron spectroscopy analysis of the poled pelletized sample (thickness: ∼0.5 mm) of **1·2H**_**2**_ **O** at room temperature. Particularly, the N_1s_ XPS spectra for both unpoled and poled samples display features characteristic of four nitrogen environments: coordinated nitrate (nitroxy), unbound nitrate, phosphoramide amido-nitrogen, and picolyl heterocyclic nitrogen. Comparison of the spectra reveals a ∼0.2 eV shift in binding energy upon poling. Deconvolution of the spectral data indicates a redistribution of populations in the poled sample: the relative intensity of the free nitrate peak decreases, while that of the coordinated nitrate (nitroxy) increases. Quantitatively, the area ratio of free nitrate to coordinated nitrate N_1s_ peaks shifts from 0.59 (unpoled) to 0.47 (poled), representing a ∼12% change in their population in favor of the coordinated nitrate anion (Figure S28).

A collective observation from the crystal structures (from 150 and 308 K), VT-Raman, XPS, and computational studies emphasizes that the polarization in the framework originates from the dynamic movement of nitrate ions. These ions initially shift along the *c*-axis and subsequently between disordered positions on either side of the 2-fold axis running through the Cu atom. Mechanistically, when an electric field is applied in a given direction along the *c*-axis, the dipole associated with the uncoordinated nitrate ion (N40 set) strengthens its interaction with the Cu­(II) ion. Subsequently, its hydrogen bond with the O1S atom weakens, facilitating the flipping of this nitrate ion along the Cu–O41 contact and allowing it to access its positionally disordered symmetry equivalent site. The contraction of the Cu–O41 distance also induces a concerted elongation of the Cu–O31 bond and concurrent flipping of the nitrate group of the N30 set to its symmetry equivalent site (Figure 4c). As the local dipole magnitude scales with the charge separation, this elongation dominates the net polarization, with the dipole vector emerging from the direction of the N30 nitrate (Figure 4a). Upon reversing the direction of the electric field, the process is inverted with the reversal of polarization (Figure 4b) as the N30 nitrate strengthens its bond with the Cu center, while the N40 nitrate recedes to its elongated position and re-establishes its H-bonding contact with the O1S at the alternative symmetry-equivalent site (Figure 4d). This reversible, distance-dependent dipole modulation confirms that the ferroelectric switching arises from the “field-induced relay” of nitrate anions. This cooperative, site-specific “anion-relay”-type ion displacement is unprecedented among reported ferroelectric systems and highlights a novel extrinsic polarization mechanism enabled by the tunable coordination chemistry of MOFs. Our findings thus introduce a new ferroelectric paradigm in which loosely bound ionic species act as active dipolar agents, offering a previously unrecognized route to electric-field-responsive behavior in low-dimensional frameworks.

### Piezoelectric Energy Harvesting Studies

Motivated by the intrinsic ferroelectric and piezoelectric properties of **1·2H**_**2**_ **O**, we investigated its potential as a piezoelectric nanogenerator (PENG) by embedding it into flexible polymer-based composite films. The composites were prepared by dispersing powdered crystals of **1·2H**_**2**_ **O** at different loadings (1, 5, 10, 15, and 20 wt %) into a homogeneous TPU solution in tetrahydrofuran (THF) (Table S6 and Figure S29). The films exhibited a uniform blue hue and demonstrated excellent mechanical flexibility and durability, retaining their structural integrity under repeated folding, rolling, bending, and twisting operations (Figure 5a and Figure S30). The PXRD patterns of the composite films confirmed the preserved crystallinity of **1·2H**_**2**_ **O** within the polymer matrix, as the diffraction peak intensities increased progressively at higher filler loadings (Figure S31). Cross-sectional SEM imaging revealed the composite film’s thickness to be approximately 0.22 mm (Figure 5a). All fabricated devices were tested under a compressive force of 21 N using a custom setup. To maximize energy harvesting, the composites were electrically poled at 6 kV/cm for 8 h, and the 10 wt %**1·2H**_**2**_ **O**–TPU device delivered the highest peak-to-peak voltage of 25.1 V (Figures 5b, 5c and S32), identifying it as the optimal composition. Notably, the trend in *V*_PP_ closely follows the piezoelectric coefficient (*d*_33_) values measured by the Berlincourt method (3.4, 16.5, 31.1, 22.7, and 5.3 pC/N for 1, 5, 10, 15, and 20 wt % composites, respectively), further validating the correlation between piezoelectric response and energy-harvesting efficiency (Figures S33 and S34). To evaluate the practical utility of the champion 10 wt %**1·2H**_**2**_ **O**–TPU device, output voltages were measured across resistances in the range of 0.1–40 MΩ, yielding a maximum instantaneous power density of 48.7 μW/cm^2^ at 0.9 MΩ (Figures 5d, S35 and S36). The device was further employed to charge commercial capacitors (10, 47, and 100 μF) via a full-wave bridge rectifier, during which the 100 μF capacitor stored 13.4 μC within 450 s, confirming its practical energy-storage capability (Figure S37). These results introduce a new class of field-tunable ferroelectric switching in coordination frameworks and offer a pathway for the design of multifunctional materials for sensing, data storage, and energy-harvesting applications.

**Figure 5:**
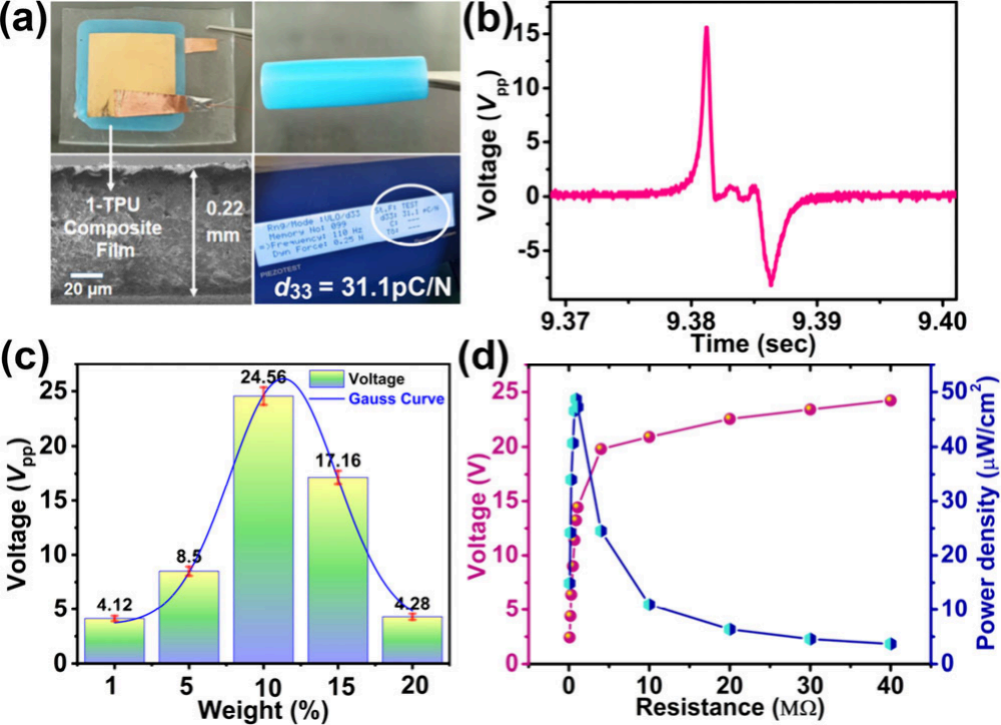
(a) Photographs of the as-prepared blue **1·2H**_**2**_ **O**–TPU composite film showing flexibility (rolled, top right), the fabricated device (top left), the cross-sectional SEM image indicating film thickness (bottom left), and the *d*_33_ meter measuring the direct piezoelectric coefficient of the 10 wt %**1·2H**_**2**_ **O**–TPU device (bottom right). (b) Output voltage plot showing a single peak from the high-performing 10 wt %**1·2H**_**2**_ **O**–TPU device. (c) Output voltage performance comparisons among 1, 5, 10, 15, and 20 wt % (**1·2H**_**2**_ **O**–TPU) composite PENG devices. The note bar represents the standard error in the output test. (d) Output voltage and power density of the 10 wt %**1·2H**_**2**_ **O**–TPU composite device under different load resistances (solid lines are a guide to the eye).

## Conclusion

In summary, we have demonstrated ferroelectricity in a Cu­(II)-based two-dimensional metal–organic framework (**1·2H**_**2**_ **O**) that features an uncommon electric-field-driven displacive switching mechanism. This “tug-of-war” polarization reversal, governed by reversible asymmetric displacement of nitrate ions, represents a previously unrecognized pathway to ferroelectricity in metal–organic frameworks. Compound **1·2H**_**2**_ **O** exhibits robust ferro- and piezoelectric responses, including a notably high direct piezoelectric coefficient (*d*_33_ = 29.4 pC/N) measured by the quasistatic “Berlincourt” method. The integration of **1·2H**_**2**_ **O** into flexible TPU-based nanogenerators further confirms its functional utility, with the best-performing device delivering an output voltage of 25.1 V and a power density of 48.7 μW/cm^2^. Though molecular ferroelectrics have been widely studied, MOF-based systems remain comparatively underexplored. The discovery of this field-tunable anion-relay mechanism broadens the spectrum of microscopic switching processes in metal–organic materials and introduces a new design strategy for multifunctional ferroelectrics. Beyond advancing the fundamental understanding, these findings lay the groundwork for rational development of MOF-based devices for energy harvesting, memory, and sensing applications.

## Supplementary Material



## Notes

Materials and methods, single crystal X-ray, powder XRD, DSC, TGA, dielectric, ferroelectric, and PFM measurements and characterization (PDF)
