# Supplementary material for: Ferroelectricity through Reversible Anion-Relay Polarization Switching in a Two-Dimensional Metal–Organic Framework
Source: J Am Chem Soc. 2026 Feb 19;148(8):8255–63. doi: 10.1021/jacs.5c18104 (PMC12964392; doi:10.1021/jacs.5c18104)
Supplement: Supplementary file 1 [file ja5c18104_si_001.pdf]

Supporting Information

Ferroelectricity through a Reversible Anion-Relay Polarization Switching in a Two-dimensional Metal–Organic Framework

Neetu Prajesh,[a] Vikash Kushwaha,[a] Chandan K. Singh,[b] Vijay Bhan Sharma,[c] Balu Praveenkumar,[d] Alexander Steiner,[e,*] Maciej Ptak,[f,*] Dinesh Kabra,[c,*] Jan K. Zaręba[g,*] and Ramamoorthy Boomishankar[a,*]

[a]Department of Chemistry and Indian Institute of Science Education and Research (IISER), Pune, Dr. Homi Bhabha Road, Pune – 411008, India
E-mail: boomi@iiserpune.ac.in

[b]Department of Physics and Indian Institute of Science Education and Research (IISER), Pune, Dr. Homi Bhabha Road, Pune – 411008, India

[c]Department of Physics, Indian Institute of Technology, Bombay, Powai, Mumbai – 400076, India
E-mail: dkabra@phy.iitb.ac.in

[d]Armament Materials & Technology Centre, Armament Research and Development Establishment (ARDE), Defence Research and Development Organisation (DRDO), Dr. Homi Bhabha Road, Pune-411021, India

[e]Department of Chemistry, University of Liverpool, Crown Street, Liverpool- L69 7ZD, United Kingdom
Email: *a.steiner@liverpool.ac.uk*

[f]Institute of Low Temperature and Structure Research, Polish Academy of Sciences, Okólna 2, Wrocław 50-422, Poland
Email: *m.ptak@intibs.pl*

[g]Institute of Advanced Materials, Faculty of Chemistry, Wroclaw University of Science and Technology, 50-370, Wroclaw, Poland
E-mail: jan.zareba@pwr.edu.pl

Table of Contents

S.No.	Contents	Page No.
1.	Experimental Section	S3-S5
2.	X-ray crystallographic Information	S6-S7
3.	Characterization data for **L** and **1**	S8-S13
4.	TGA, DSC, Adsorption Isotherm, and cell parameters analysis of dehydrated and hydrated **1**	S13-S14
4.	VT-PXRD, SHG data and dielectric data	S15-S17
5.	Point-charge calculations	S18-S19
5.	Fatigue data, PFM, d_{33}, Computational and XPS studies of **1·2H₂O**	S19-S22
6.	Characterization and testing of **1·2H₂O**-TPU composite films	S22-S29
7.	References	S29-S30

Experimental Section

General remarks. All operations involving phosphorus halides were carried out under a dry nitrogen atmosphere using standard Schlenk techniques. Toluene was dried over sodium prior to use. 3-Picolylamine was purchased from Aldrich and used without further purification. $PhPOCl_2$ and $Cu(NO_3)_2{\cdot}3H_2O$ were obtained from Acros Organics and Merck, respectively, and used as received. NMR spectra were recorded at room temperature on a Bruker 400 MHz spectrometer (^1H NMR: 400.13 MHz; ^{13}C NMR: 100.62 MHz; $^{31}P\{^1H\}$ NMR: 161.97 MHz), using tetramethylsilane (TMS) as an internal standard for ^1H and ^{13}C, and 85% H_3PO_4 as an external standard for ^{31}P. Powder X-ray diffraction (PXRD) patterns were collected using a Bruker D8 Advance diffractometer. Thermogravimetric analysis (TGA) was conducted on a PerkinElmer STA-6000 analyzer. Elemental compositions were determined using a Vario EL Cube elemental analyzer. Melting points were measured with an Electrothermal apparatus and are reported without correction. Temperature-dependent Raman spectra were measured using a Renishaw inVia Raman spectrometer operating at 514.5 nm and combined with a Linkam THMS600 stage. Differential scanning calorimetry (DSC) measurements were performed on a TA Q20 differential scanning calorimeter (DSC) at heating and cooling rates of 10 °C/min under a nitrogen atmosphere.

Synthesis. L: To a stirred solution of 3-picolylamine (4.575 g, 0.0423 mol) in toluene (~110 mL) at 0 °C, $PhPOCl_2$ (1 mL, 1.375 g, 0.00705 mol) in 10 mL of toluene was added dropwise through a pressure equalizer funnel under an inert atmosphere, and the reaction mixture was refluxed for 4 h. The toluene solution was subsequently filtered, and the obtained white precipitate was washed with dichloromethane three times. The filtrate and washings were evaporated under reduced pressure to yield a white precipitate of L, which was then dried and collected. Yield: 1.56 g (79.18%). M.P.: 105-110 °C. ^1H NMR ($CDCl_3$): δ 8.45 (d, 2H, CH(pyridyl)), δ 8.4 (dd, 2H, CH(pyridyl)), δ 7.6 (td, 2H, CH (pyridyl)), δ 7.12 (dd, 2H, CH (pyridyl)), δ 3.44 (t, 2H, NH), δ 4.1 (d, 4H, CH_2), δ 7.43 (1H, phenyl), δ 7.5 (2H, phenyl), δ 7.8 (2H, phenyl). $^{13}C\{^1H\}$ NMR ($CDCl_3$): δ 42.3, 123.5, 128.7, 131.6, 132, 135.3, 148.7, 148.9. ^{31}P NMR (DMSO-d_6): δ 21.14. Anal. Calcd. for $C_{18}H_{19}N_4OP$: C, 63.9; H, 5.66; N, 16.56. Found: C, 63.5; H, 5.44; N, 16.12.

1·3H$_2$O: To a stirred solution of **L** (30 mg, 0.088 mmol) in MeOH (3mL), $Cu(NO_3)_2{\cdot}3H_2O$ (14 mg, 0.057 mmol) in H_2O (1 mL) was added. The resulting solution was subsequently stirred for 2 h and filtered through celite pad. The filtrate was left for crystallization. The suitable blue crystals for X-ray analysis were obtained after 2-3 weeks. Yield: 45.4% (23.6 mg), based on Cu. Calcd. for $C_{36}H_{44}N_{10}O_{11}P_2Cu$: C, 47.09; H, 4.83; N, 15.25. Found: C, 47.32; H, 4.60; N, 15.27.

1·2H$_2$O: Drying of crystalline **1·3H$_2$O** in a vacuum desiccator resulted in the formation of **1·2H$_2$O**. Calcd. for $C_{36}H_{42}N_{10}O_{10}P_2Cu$: C, 48.03; H, 4.70; N, 15.56. Found: C, 48.12; H, 4.62; N, 15.52.

Crystallography: Reflections were collected on a Bruker Smart Apex Duo diffractometer at 120 K (for L), 150 K (for **1·3H$_2$O**), and 308 K (for **1·2H$_2$O**) using MoKα radiation (λ = 0.71073 Å). The 298 K and 150 K data for **1·2H$_2$O** were obtained after annealing the crystal at 340 K for 1 hour. Structures were refined by full-matrix least squares against F^2 using all data using SHELX.[1] All non-hydrogen atoms were refined anisotropically if not stated otherwise. In all four structures, the nitrate ions are disordered about the crystallographic 2-fold axis. These were refined with restraints for all structures and isotropically for those of

1·2H_2O. Crystals of **1·2H_2O** that were subjected to drying treatment suffered increased mosaicity, resulting in a loss in resolution; hence, their data were truncated at 1.00 Å. The disordered fragments were refined with similar distances and U-restraints by using the SIMU/SAME command in Shelx.

Second Harmonic Generation Study: Kurtz–Perry powder tests were performed using a femtosecond laser setup. The system was based on a Coherent Astrella Ti:sapphire regenerative amplifier (RA) generating ultrashort pulses (800 nm, 75 fs) at a repetition rate of 1 kHz. The unfocused output of the RA was directed onto the samples and attenuated using a neutral-density reflective filter, resulting in a laser fluence of 0.20 mJ cm^{-2}. The relative SHG efficiency of **1·2H_2O** was estimated using the Kurtz–Perry (Graja) method,[2,3] with potassium dihydrogen phosphate (KDP) serving as the reference material (see Figure S12). **1·2H_2O** and KDP crystals were ground and sieved using an Aldrich mini sieve set to obtain a 177–125 μm microcrystal fraction. The sieved samples were sealed and mounted in the sample holder between tightly pressed microscope glass slides. The laser beam was incident on the samples at 45° and remained unfocused in all cases. SHG signals were collected in backscattering geometry, with optics coupled to a glass optical fiber placed perpendicular to the sample plane. Scattered fundamental radiation was suppressed with a 750 nm hard-coated short-pass dielectric filter (FESH0750, Thorlabs). The diffuse SHG spectra were recorded using an Ocean Optics Flame T XR fiber-coupled CCD spectrograph with a 200 μm entrance slit. VT-SHG measurements using the same signal collection geometry and temperature control of the sample were performed using a Linkam LTS420 Heating/Cooling Stage. Temperature stability was equal to 0.1 K.

Ferroelectric and Dielectric Measurements: To determine the dielectric properties, a powdered sample of **1·2H_2O** was compacted into a disc (approximately 13 mm in diameter and 0.131 mm in thickness). The compacted discs were subsequently electroded using silver paint for both measurements. The frequency-dependent dielectric characteristics for **1·2H_2O** at various temperatures were measured using the Novocontrol dielectric spectrometer.

The *P–E* hysteresis loop measurement was performed on the thin film of **1·2H_2O** using a Sawyer-Tower circuit at room temperature. The thin film was prepared by the drop-casting method: 20 mg of **1** was dissolved in 0.5 ml of methanol and dropped onto the ITO-coated glass. The blue-coloured micrometer-sized crystallites of **1·2H_2O** were observed on the ITO substrate after one hour. The film was allowed to dry at ambient conditions in a desiccator. The conductive indium-gallium alloy was used as electrodes and applied on the ITO substrate and on the thin film to function as the bottom and top electrodes. The ferroelectric measurements were performed on an aixACCT TF2000E model hysteresis loop analyser. The hysteresis loop was obtained using the double-wave method, employing the positive up and negative down (PUND) function of the instrument at 20 Hz frequency. Leakage currents were collected dynamically for various voltage steps during the hysteresis loop measurements.

Piezoresponse Force Microscopy Characterization: Piezoelectric characterizations of **1·2H_2O** were studied under the Asylum Research MFP-3D atomic force microscopy (AFM) system. The contact mode AFM (c-AFM) was used to obtain piezoresponse of the films. The measurement was performed using RMN–12PT300B cantilever probes with a spring constant of 1.12 N/m and a tip diameter of <8 nm. The PFM data collected were based on the vertical PFM measurements (VPFM), where the DC voltage was applied to the conductive AFM tip, keeping the bottom electrode grounded. The PFM images were collected at a resonance frequency of 300 +/– 20 kHz with an applied AC bias of 40 V and an AC bias 2.5 V. The measurements were performed in dual ac resonance tracking (DART) mode of the PFM.

X-ray Photoelectron Spectroscopy (XPS): The experiments on sample **1·2H_2O** were performed using a monochromatic Al K_α X-ray source (E_{ex} = 1486.0 eV) with an EA15 hemispherical Electron Energy Analyser, manufactured by PREVAC (Poland). The spectral acquisition was carried out in normal emission geometry with a pass energy of 200 eV. The X-ray source was operated at a power of 240 W and in the presence of a continuous flood (electron) source (Energy = 10.0 V; $I_{emission}$ = 10.0 μA). The measurements were conducted on thin pelletized samples. Two identical pellets were prepared: one was used directly for characterization, while the second pellet was subjected to corona poling at 25 kV for approximately 6 hours. After the poling process, this poled pellet was subsequently used for the corresponding measurements. The samples were loaded on carbon conductive tape, and the binding energies (BE) of elements were calibrated with respect to C_{1s} photoemission at 284.8 eV. During the measurement process, identical experimental conditions were maintained for both pelletized samples to ensure that any spectral differences observed could be attributed solely to the poling treatment. XPS spectra were recorded at 304 K while maintaining the base pressure of the analyser chamber better than 1 × 10^{-8} mbar.

Low-pressure water adsorption studies: Low-pressure water adsorption–desorption measurements were carried out using a BelAqua instrument (BEL Japan). The as-synthesized **1·2H_2O** powder was preheated at 353 K under vacuum for 24 h to activate the **1·2H_2O**, and the absence of desolvation was confirmed by TGA analysis. Prior to the water adsorption measurements, the **1·2H_2O** sample was further pre-treated at 353 K under vacuum for 4 h in the BelAqua system and then cooled to room temperature under a nitrogen purge.

Computational Details: First-principles calculations were performed using Density Functional Theory (DFT)[4] with the PBEsol functional[5] and a plane–wave basis set, as implemented in the Quantum ESPRESSO package.[6] A simplified model of the 2D MOF crystal structure was used to explore the origin of ferroelectricity and to analyze interactions among metal ions, ligands, and nitrate groups. The unit cell contains 440 atoms, requiring substantial computational effort. The system was placed in a 35 × 30 × 30 $Å^3$ simulation box with a 20 Å vacuum layer to avoid periodic interactions. An external electric field was applied along the z-direction using a sawtooth potential.[7] The dipole moment was calculated using a Python script that evaluates the induced dipole moment of the molecule within a 35 Å cubic supercell. The calculation is based on the differential charge density (Δρ) obtained from *Quantum ESPRESSO* (QE) pp.x outputs,[6] which were exported as Gaussian cube files using VESTA. The induced dipole moment is calculated as:

$$\mu = \int r\,\Delta\rho(r)\,dV$$

where Δρ(r) = ρ(with electric field) − ρ(without electric field) is the differential charge density, integrated over the 35 Å cubic cell with positions r relative to the box center.

Table S1: Crystallographic data for the ligand **L**, **1·3H_2O (150 K)** and **1·2H_2O (308 K)**.

**Compound**	**L**	**1·3H_2O (150 K)**	**1·2H_2O (308 K)**
Chemical formula	$C_{18}H_{19}N_4OP$	$C_{36}H_{44}N_{10}O_{11}P_2Cu$	$C_{36}H_{42}N_{10}O_{10}P_2Cu$
Formula weight	338.34	916.27	896.24
Temperature	120 K	150 K	308 K
Crystal system	Orthorhombic	Orthorhombic	Orthorhombic
Space group	$Pca2_1$	*Aba*2	*Aba*2
a (Å); α (°)	16.208(3), 90	16.424(3), 90	16.483(8), 90
b (Å); β (°)	5.3177(9), 90	23.743(5), 90	23.710(11), 90
c (Å); γ (°)	38.178(7) Å, 90	10.653(2), 90	10.430(5), 90
V (Å^3); Z	3290.5(10); 8	4154.2(14); 4	4076(3); 4
ρ (calc.) Mg m^{-3}	1.366	1.465	1.467
μ (Mo Kα) mm^{-1}	0.180	0.673	0.683
$2\theta_{max}$ (°)	40.778	50.05	50.048
R(int)	0.0731	0.1175	0.1331
Completeness to θ	100 %	100 %	99.9 %
Flack parameter	0.04(7)	-0.01(3)	0.027(16)
Data / param.	3238/433	3610/319	3609/283
GOF	1.127	1.027	1.041
R_1 [F>4σ(F)]	0.0394	0.0567	0.0596
wR_2 (all data)	0.1038	0.1301	0.1872
max.peak/hole (e.Å^{-3})	0.239/-0.268	0.302/-0.398	0.554/-0.429

Table S2: Crystallographic data for **1·2H$_2$O (150 K)** and **1·2H$_2$O (298 K)**.

**Compound**	**1·2H$_2$O (150 K)**	**1·2H$_2$O (298 K)**
Chemical formula	$C_{36}H_{42}N_{10}O_{10}P_2Cu$	$C_{36}H_{42}N_{10}O_{10}P_2Cu$
Formula weight	900.27	900.27
Temperature	150 K	298 K
Crystal system	Orthorhombic	Orthorhombic
Space group	*Aba*2	*Aba*2
a (Å); α (°)	16.311(9), 90	16.504(6), 90
b (Å); β (°)	23.627(14), 90	23.715(9), 90
c (Å); γ (°)	10.300(6), 90	10.430(4), 90
V (Å^3); Z	3969(4); 4	4082(3); 4
ρ (calc.) Mg m^{-3}	1.506	1.465
μ (Mo Kα) mm^{-1}	0.701	0.682
$2\theta_{max}$ (°)	41.63	41.62
R(int)	0.1990	0.3378
Completeness to θ	99.9 %	99.9 %
Flack parameter	0.84(10)	0.20(10)
Data / param.	2033/264	2145/264
GOF	1.060	1.070
R_1 [F>4σ(F)]	0.0887	0.0910
wR_2 (all data)	0.2141	0.2223
max.peak/hole (e.Å^{-3})	0.764/-0.514	0.593/-0.371

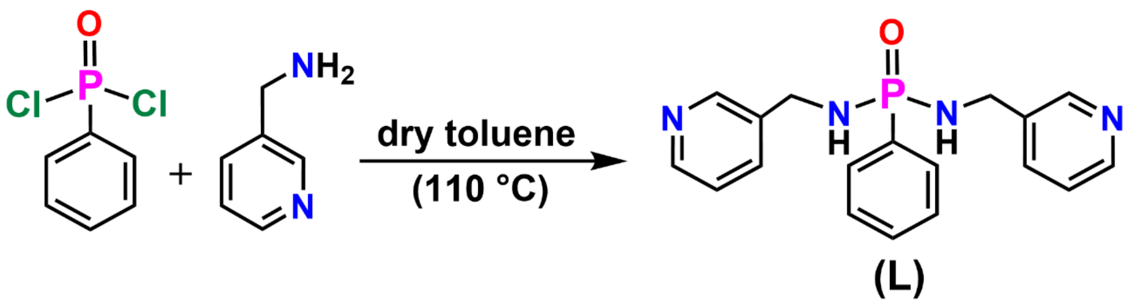

Scheme S1: Synthesis of ligand **L**.

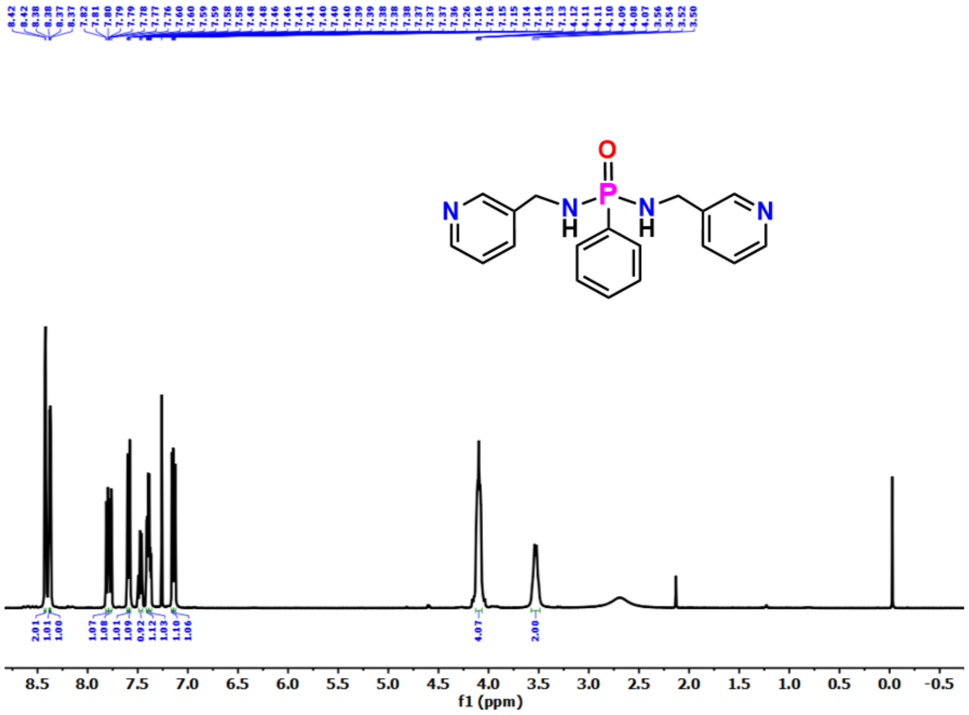

Figure S1: The ^{1}H NMR spectrum of **L**.

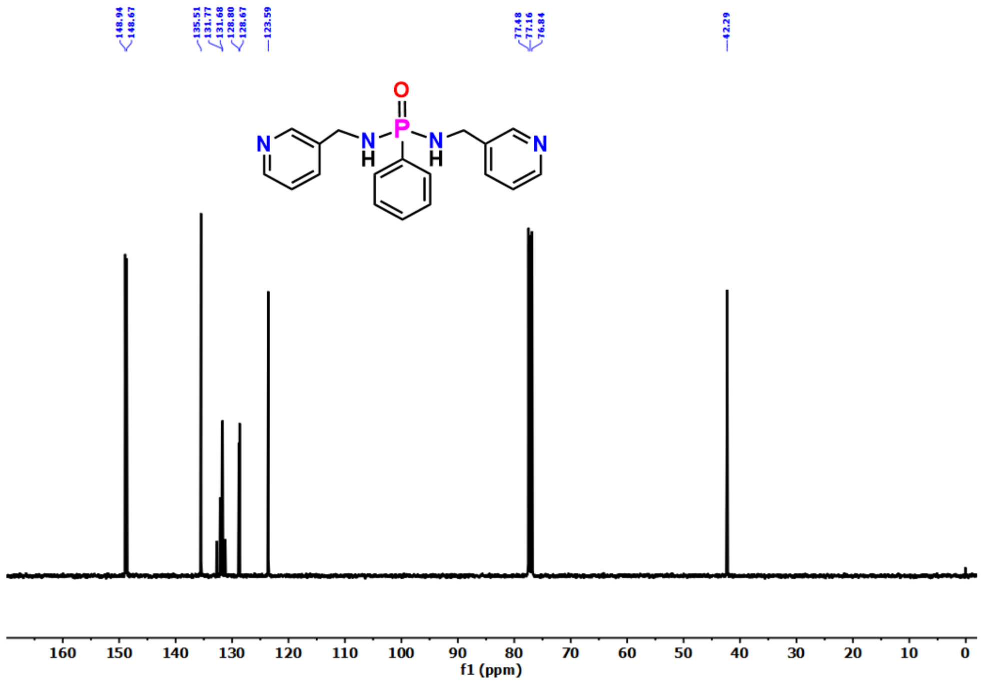

Figure S2: The ^{13}C NMR spectrum of **L**.

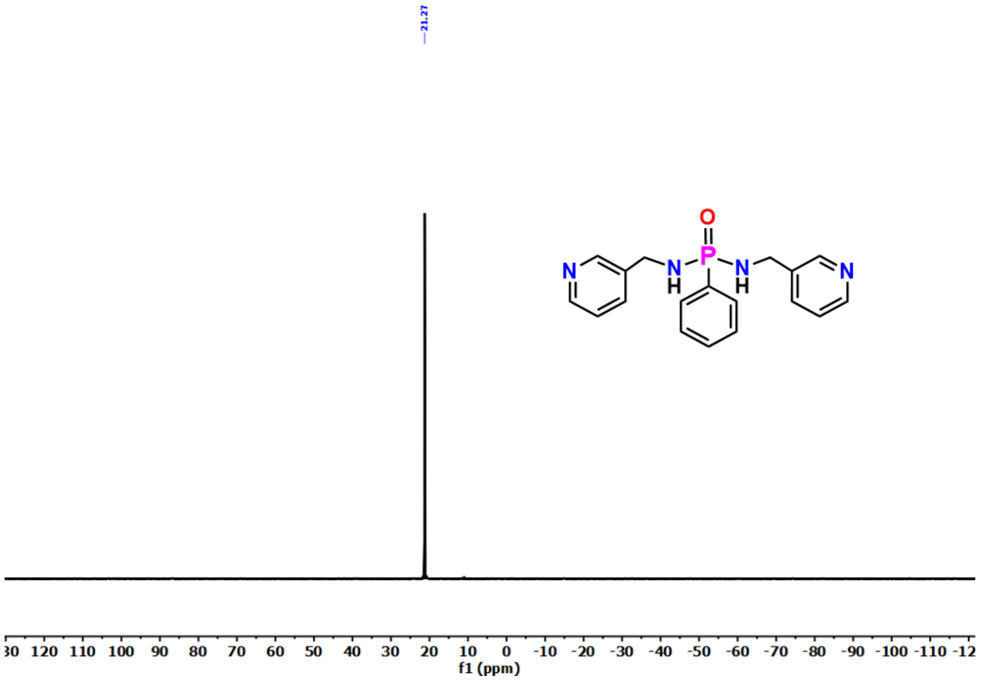

Figure S3: The $^{31}P\{^1H\}$ NMR spectrum of **L**.

Crystal structure of L

The molecular structure of the ligand $PhPO(NHCH_2(^3Py))_2$ (**L**) was solved in the orthorhombic non-centrosymmetric polar space group $Pca2_1$. The asymmetric unit contains two molecules of the ligand. In the crystal packing, one amino arm participates in N–H···O hydrogen bonding

with the phosphoryl oxygen of a neighboring ligand, while the second amino arm engages in N–H···N hydrogen bonding with the pyridyl nitrogen of another ligand, resulting in a hydrogen-bonded network.

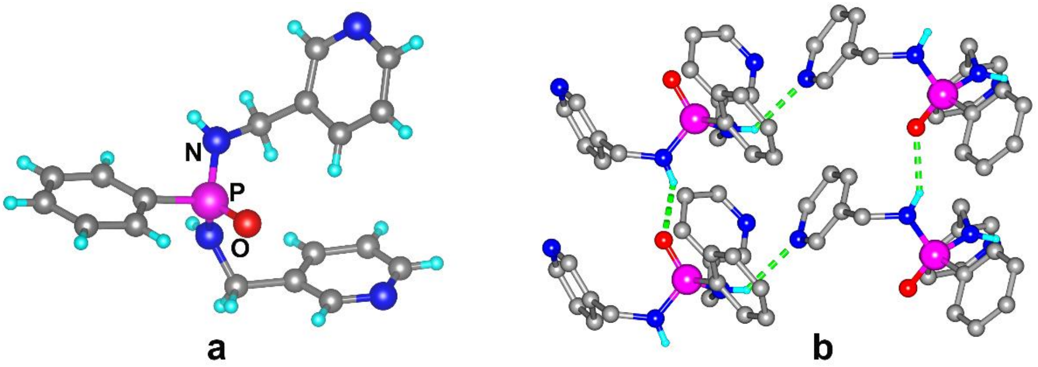

Figure S4: Crystal structure of the ligand **L** (a) View of the monomeric unit of **L** (b) The hydrogen bonding interactions involving the N-H···O=P and N-H···N_{Py} units.

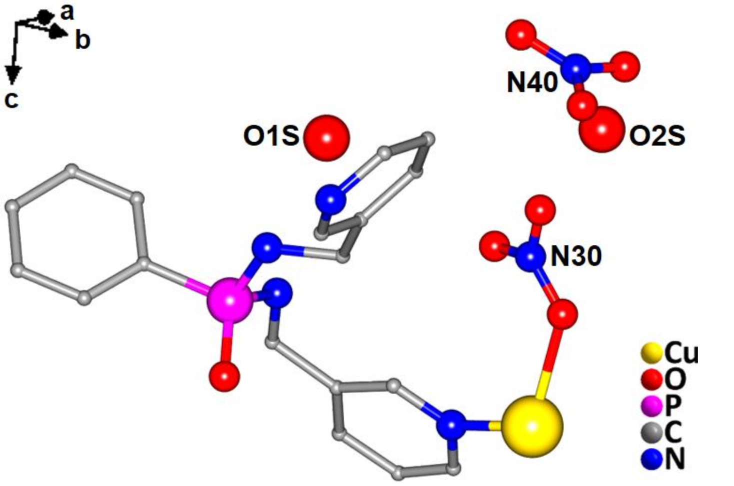

Figure S5: Asymmetric unit of **1·3H_2O** at 150 K.

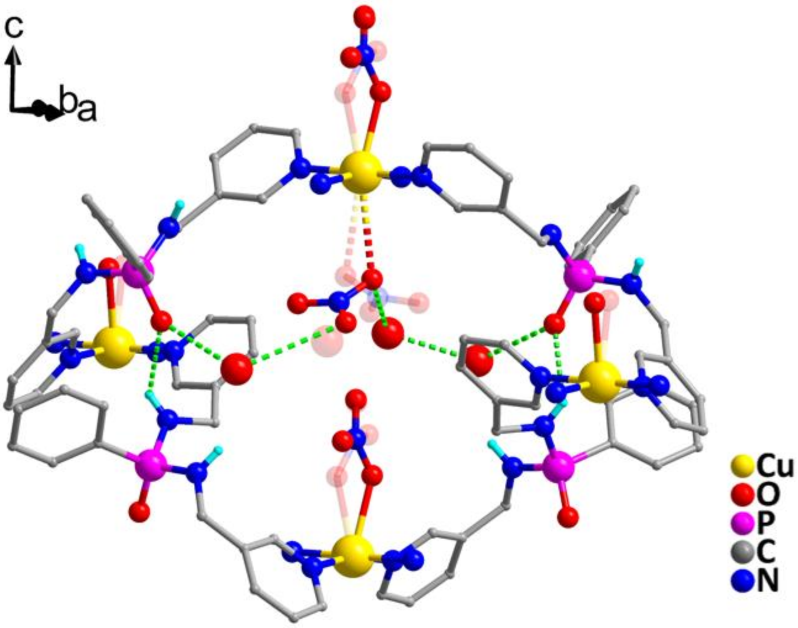

Figure S6: Fragment of the crystal structure of **1·2H$_2$O** at 308 K showing the coordination environment forms a channel in which water molecules form hydrogen bonds with phosphoramide oxygen (O–H···O=P) and simultaneously accepts a hydrogen bond from the amide nitrogen (O···H–N) of the ligand while also forming an interaction with one of the nitrate ions.

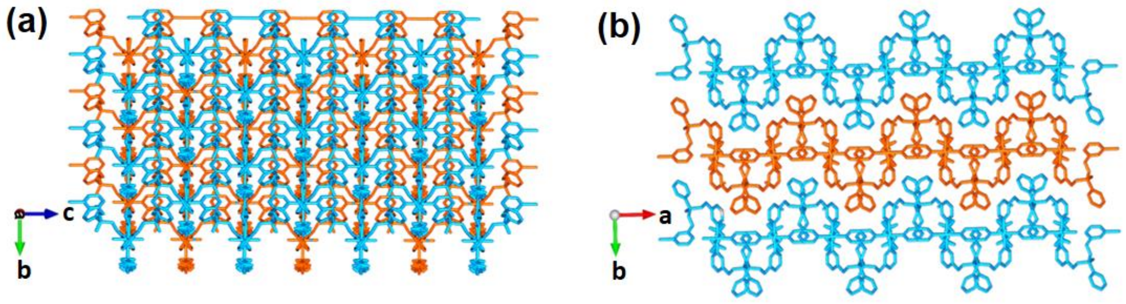

Figure S7: Packing structure of compound **1·3H$_2$O** viewed along the *a*- (a) and *c*-axes (c) at 150 K.

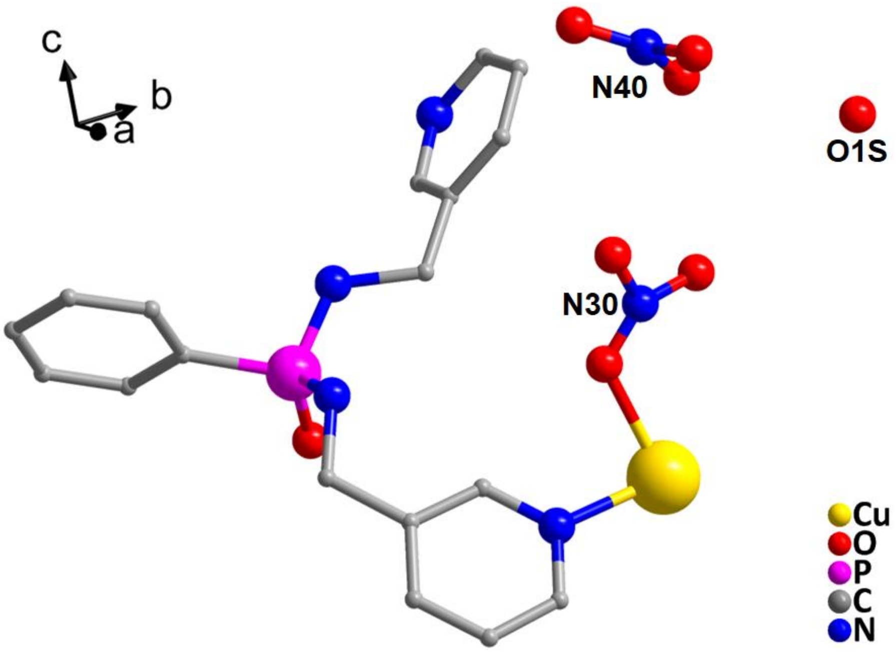

Figure S8: Asymmetric unit of **1·2H_2O** at 308 K.

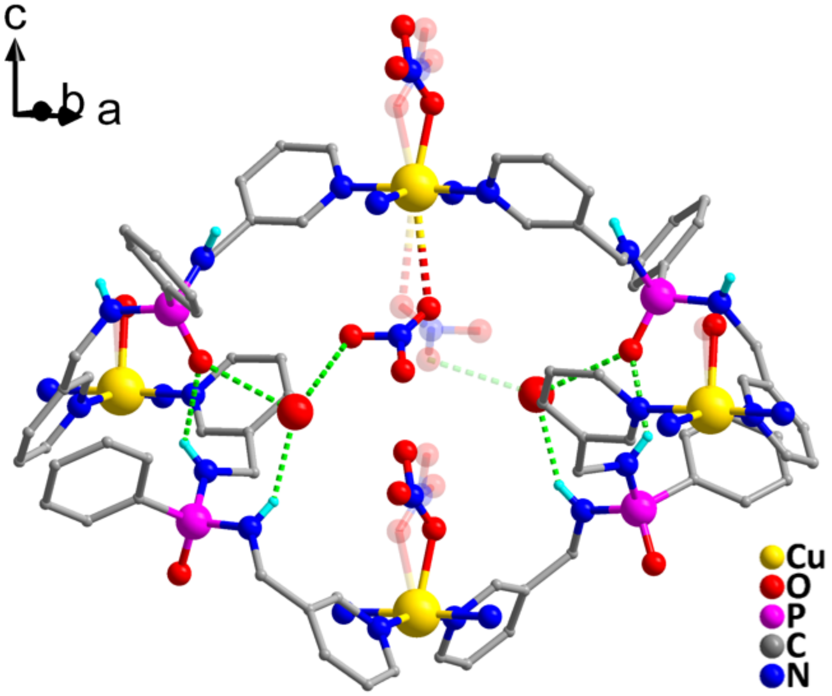

Figure S9: Fragment of the crystal structure of **1·2H_2O** at 308 K showing the coordination environment forms a channel in which water molecules form hydrogen bonds with the disordered nitrate anions and phosphoryl oxygen atoms of the ligand.

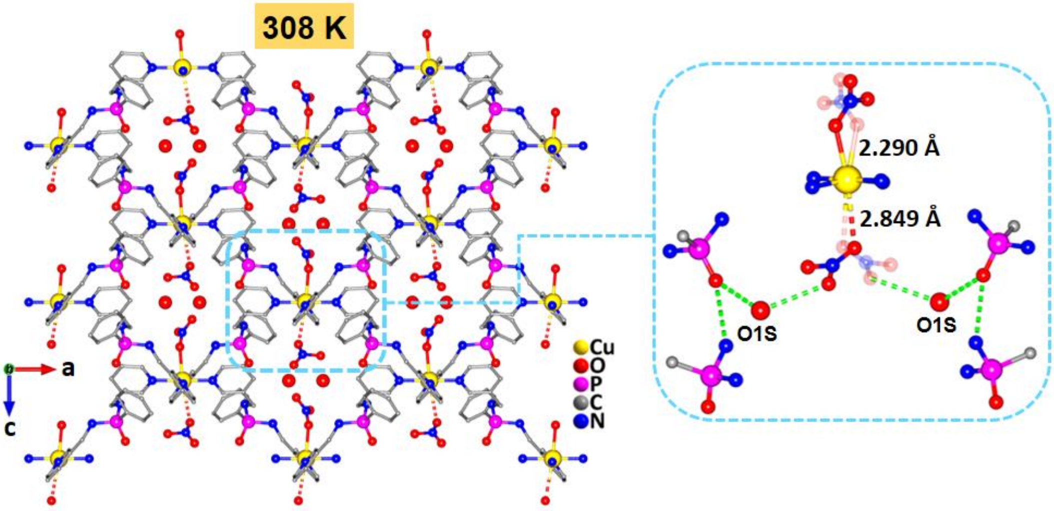

Figure S10: View of the 2D-framework in **$1\cdot2H_2O$** at 308 K. The inset shows a zoomed-in view of the Cu(II)-nitrate interactions.

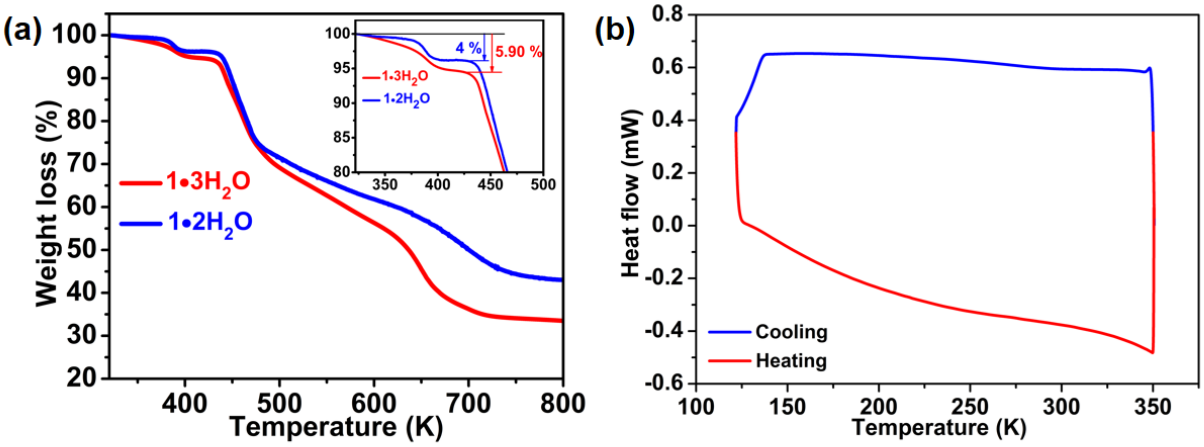

Figure S11: (a) Thermogravimetric data of **$1\cdot3H_2O$** and **$1\cdot2H_2O$** (inset: closer view of the desolvation profile). (b) Differential scanning calorimetry (DSC) of **$1\cdot2H_2O$**

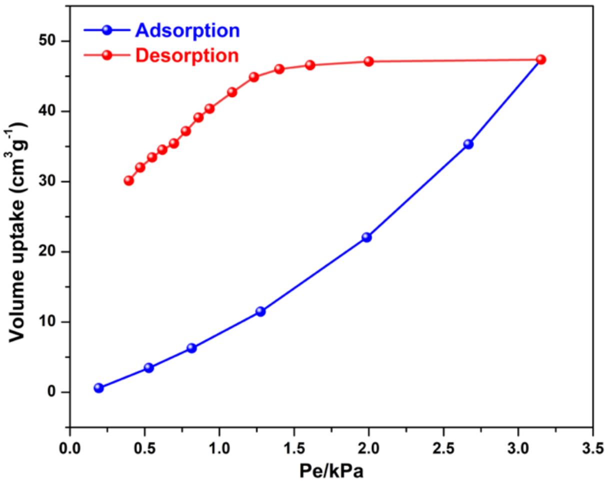

Figure S12: Water adsorption isotherm of **1·2H_2O** showing the adsorption and desorption profiles**.**

Table S3: Unit cell parameters of **1·3H_2O** and **1·2H_2O** drawn from single-crystal X-ray diffraction solution at different temperatures.

Sample		a (Å)	b (Å)	c (Å)	V (Å)^3
**1**	**1·3H_2O (150 K)**	16.424(3)	23.743(5)	10.653(2)	4154.2(14)
**2**	**1·2H_2O (308 K)**	16.483(8)	23.710(11)	10.430(5)	4076(3)
**3**	**1·2H_2O (298 K)**	16.504(6)	23.715(9)	10.430(4)	4082(3)
**4**	**1·2H_2O (150 K)**	16.311(9)	23.627(14)	10.300(6)	3969(4)

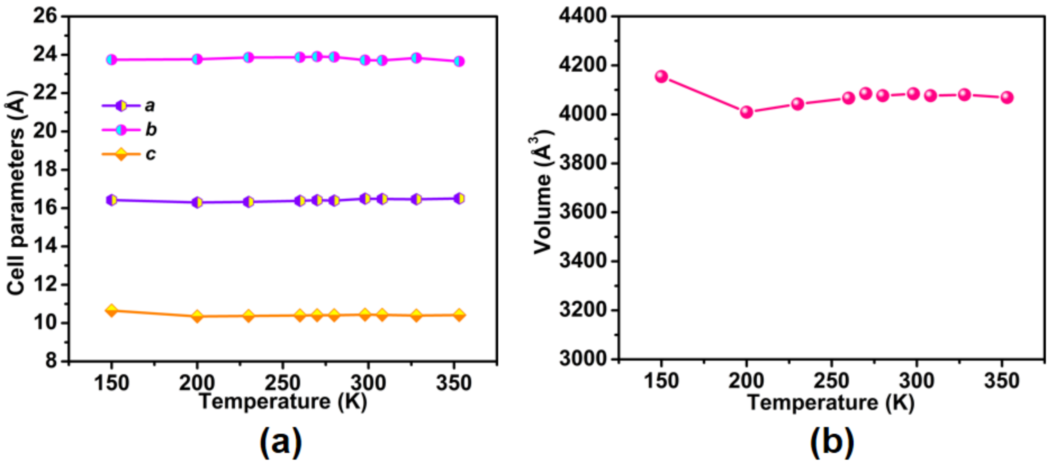

Figure S13: Temperature dependence of **1·3H_2O** showing the unit cell parameters between 150 K and 353 K. (a) cell axes (b) Unit cell volume.

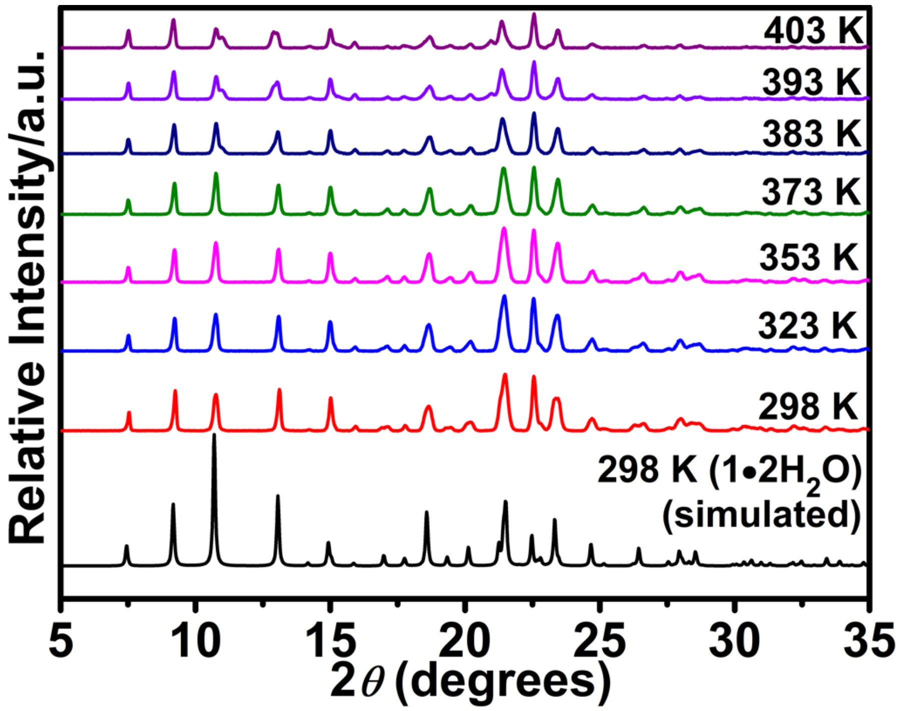

Figure S14: Variable-temperature powder X-ray diffraction (VT-PXRD) profiles of **1·2H₂O** in the temperature range between 298 K and 403 K.

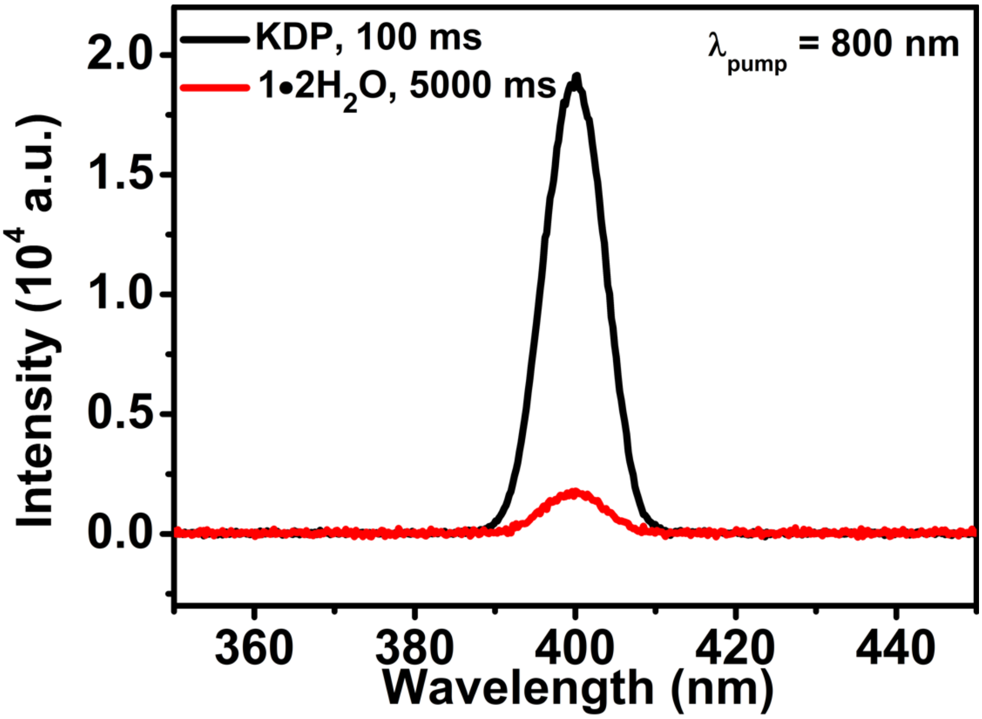

Figure S15: The SHG signal of **1·2H₂O** at room temperature (RT) overlaid with that of KDP of the same particle size.

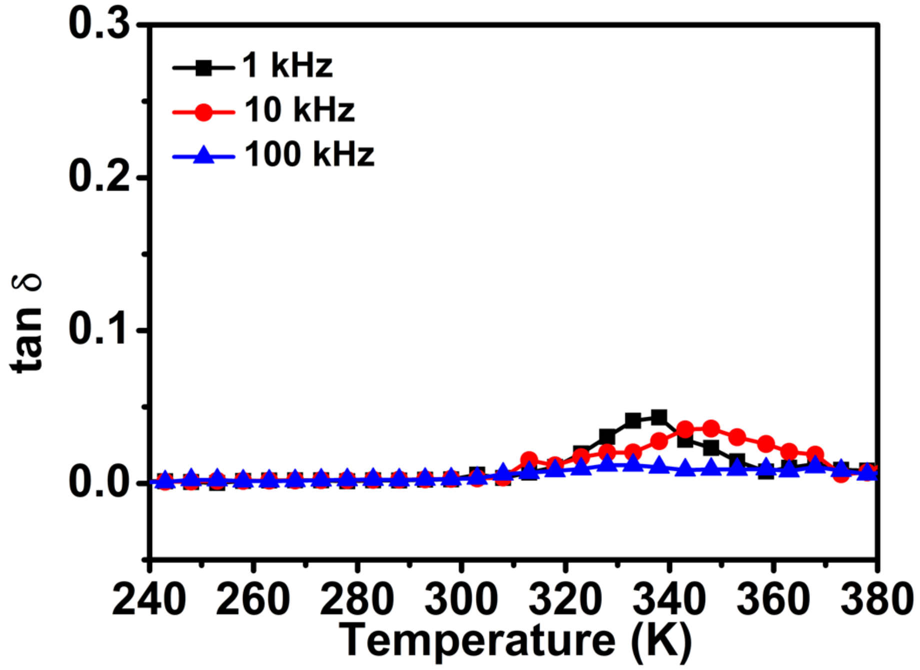

Figure S16: Dielectric loss factor (tan δ) of **1·2H_2O** as a function of temperature.

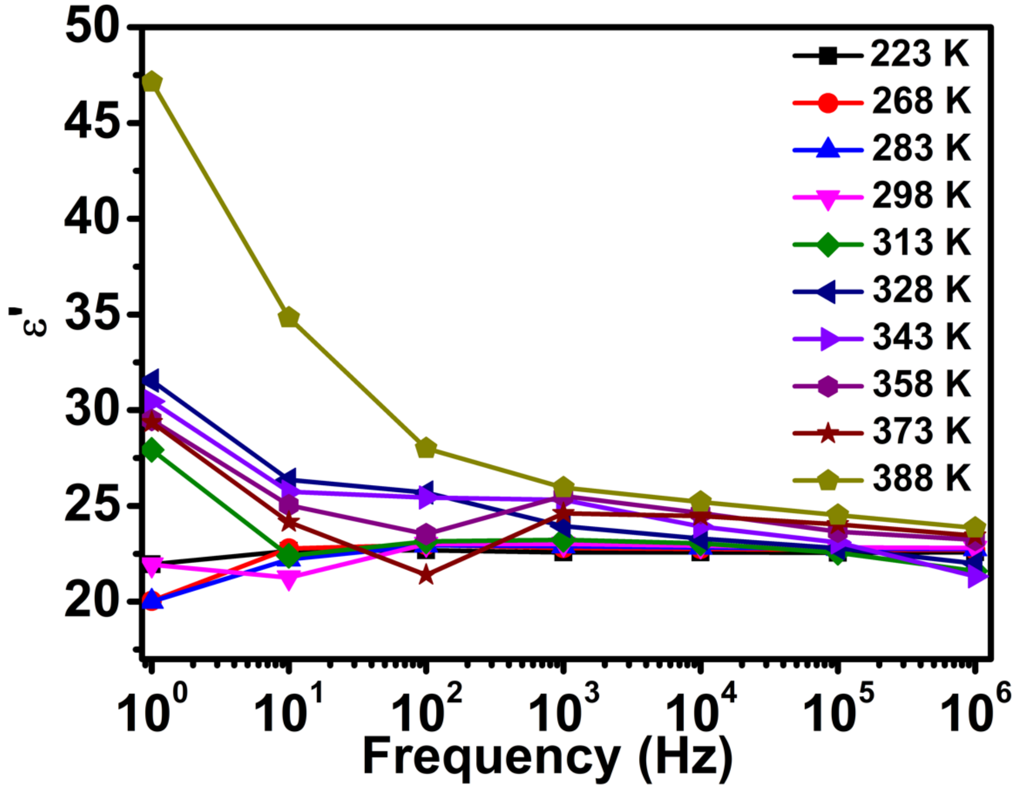

Figure S17: Real part of the dielectric permittivity (ε') graph of **1·2H_2O** as a function of frequency.

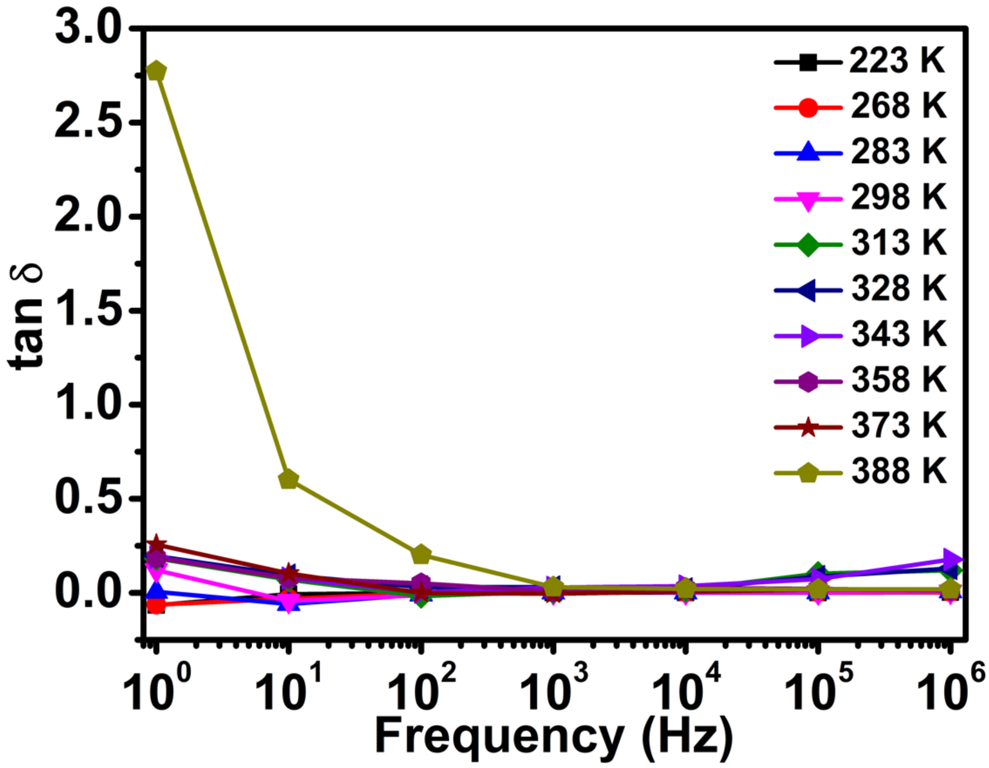

Figure S18: Dielectric loss factor (tan δ) of **1·2H_2O** as a function of frequency.

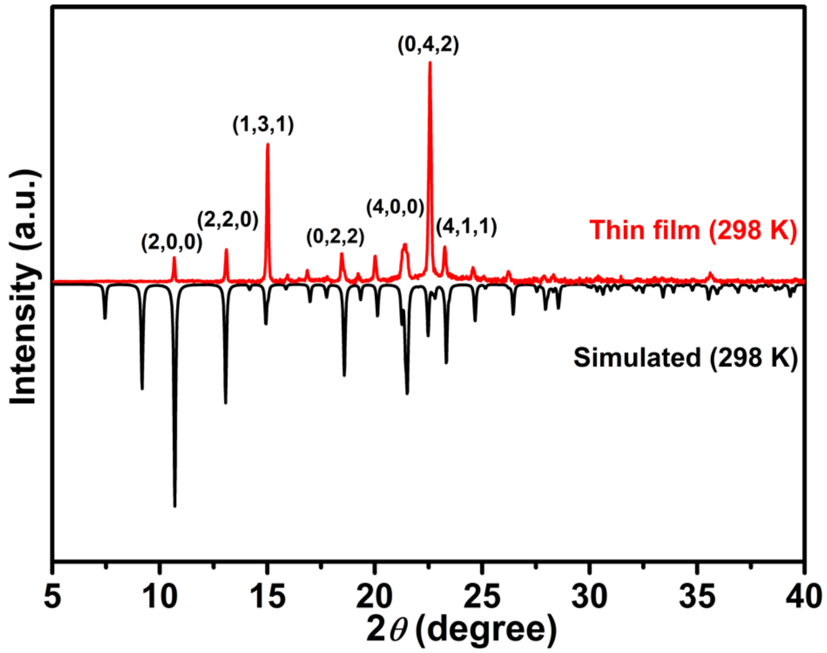

Figure S19: Powder X-ray diffraction pattern for the thin film of **1·2H_2O** and its comparison with the simulated pattern from the X-ray-derived structure at 298 K.

Point charge model polarization calculation for 1·2H_2O:

According to the crystal structure of **1·2H_2O** at 308 K, we select a unit cell and make an assumption that the positive point charges on the Cu^{2+} ions, and the negative charges at the averaged coordinates of the two disordered nitrate oxygen positions, representing the effective position of the NO_3^- anion. The charge distribution is listed in Table S3.

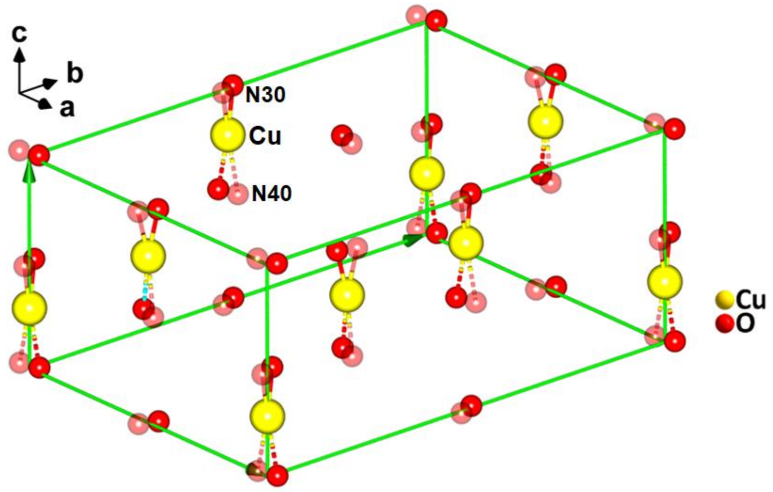

Figure S20: Unit cell of **1·2H_2O** at 308 K showing the Cu^{2+} atoms and the disordered nitrate oxygen positions used for the point-charge calculation.

Table S4: Charge distribution of the unit cell of **1·2H_2O**.

Atoms	Fractional Coordinates	Centroid Coordinates
**Cu**	(0, 0, 0.28513) (0.0, 0.5, 0.78513) (0.5, 0.0, 0.78513) (0.5, 0.5, 0.28513)	(0.25, 0.25, 0.5350)
**N30**	(0, 0, 0.50005) (0.5, 0, 0.00005) (0, 0.5, 0.00005) (0.5, 0.5, 0.50005)	(0.25, 0.25, 0.25005)
**N40**	(0, 0, 0.01641) (0, 0.5, 0.51641) (0.5, 0.5, 0.01641) (0.5, 0, 0.51641)	(0.25, 0.25, 0.26641)

		Cartesian Coordinates			
**Atom**	**Charge**	**x (Å)**	**y (Å)**	**z (Å)**	**q·r (e·Å)**
**Cu**	+2	4.127	5.914	5.578	(8.254, 11.828, 11.156)
**N30**	-1	4.127	5.914	2.6057	(−4.127, −5.914, −2.6057)
**N40**	-1	4.127	5.914	2.776	(−4.127, −5.914, −2.776)

$P_s = lim \frac{1}{V} \sum qiri$

$= (q_{Cu} r_{Cu} + q_{N30} r_{N30} + q_{N40} r_{N40}) / V$

$= [(11.156) + (-2.6057) + (2.776)] / V$

$= [(5.7743) \times 1.602 \times 10^{-19} \times 10^{-10}\ C\ m] / (4068.91 \times 10^{-30}\ m^3)$

$= 0.02273\ C/m^2$

$|P_s| = (0, 0, 2.27)\ \mu C/cm^2$

The calculated spontaneous polarization is primarily along the crystallographic c-axis. The net dipole components along a and b axes are zero due to symmetry, while the nonzero c-component results in:

$|P_s| \approx 2.27\ \mu C/cm^2$

This indicates negligible polarization along x and y directions, confirming that the polarization is essentially one-dimensional along z.

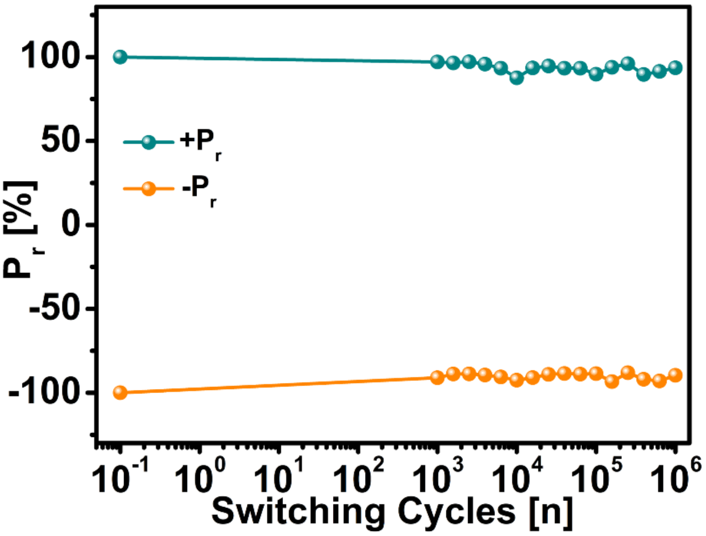

Figure S21: Ferroelectric fatigue profile data of **1·2H$_2$O** up to 10^6 cycles.

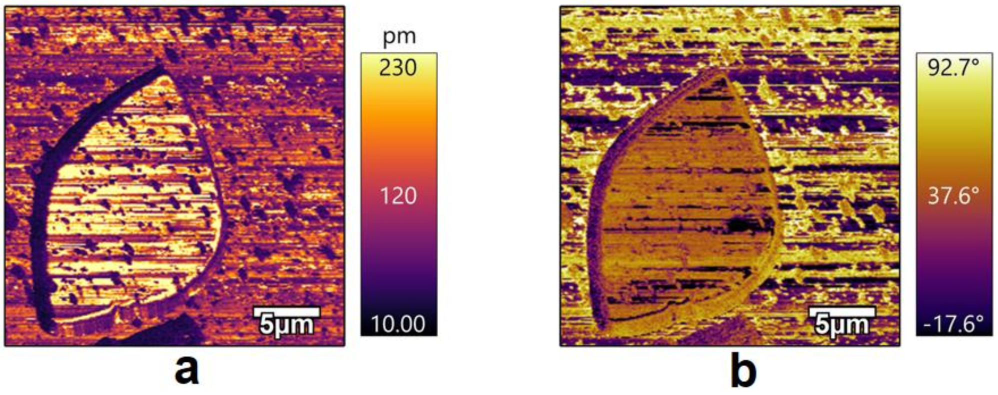

Figure S22: Vertical PFM data of **1·2H$_2$O**. (a) amplitude and (b) phase images for a 30 x 30 μm region in the as-grown single crystalline thin film.

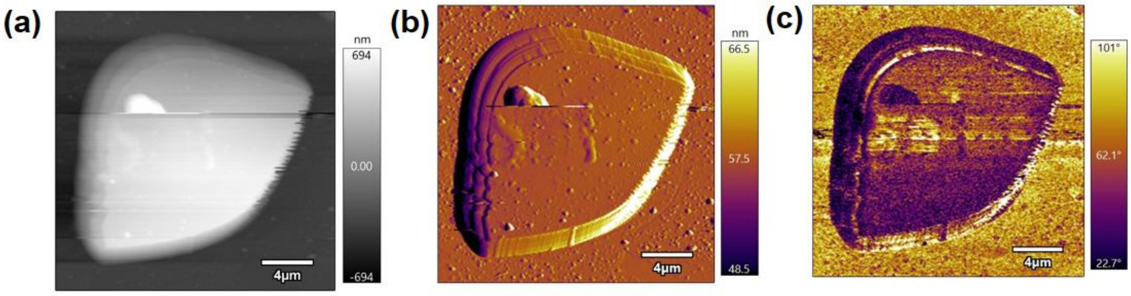

Figure S23: Vertical PFM data of **1·2H$_2$O** at a second location of the thin-film showing (a) topography, (b) amplitude, and (c) phase images.

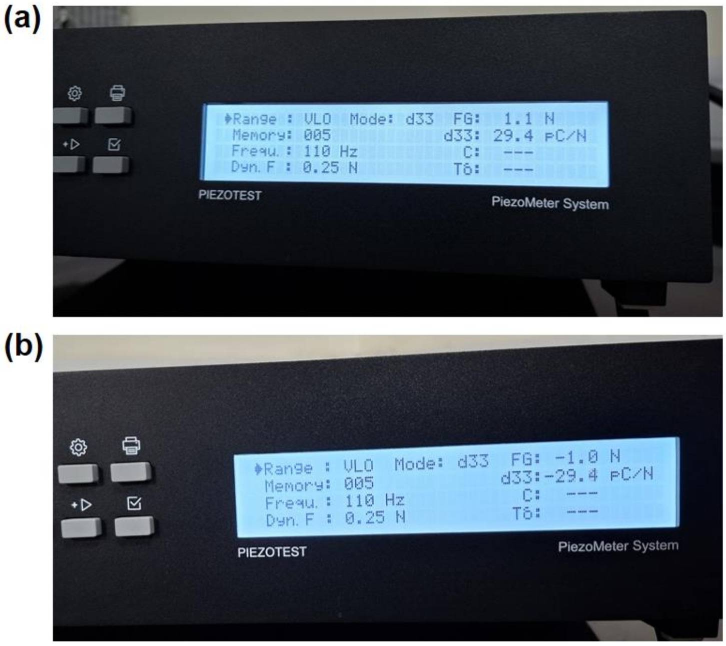

Figure S24: Piezoelectric coefficient (d_{33}) of the electrically poled pelletised sample of **1·2H$_2$O** subjected to a poling field of 25 kV. (a) Measured d_{33} value in the forward loading direction under an applied force of 1.1 N. (b) Measured d_{33} value in the reverse loading direction under an applied force of 1.0 N.

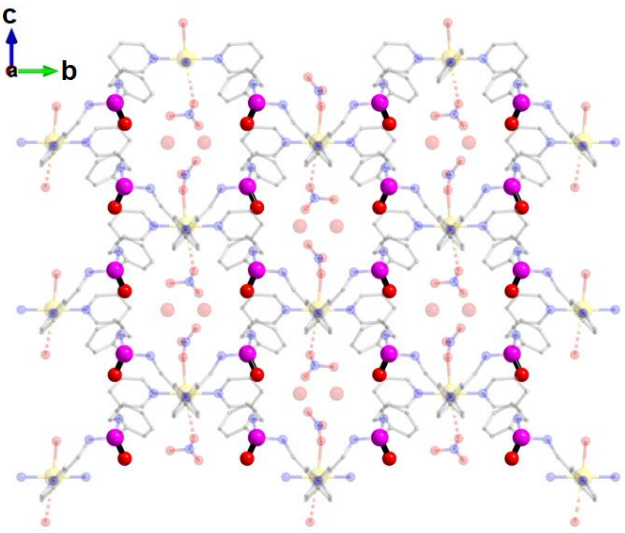

Figure S25: View of the cationic 2D network in **1·2H_2O** showing the rigid polar Cu(II)–phosphoramide framework with all the P=O units aligned in one direction. Note: P: magenta and O: red.

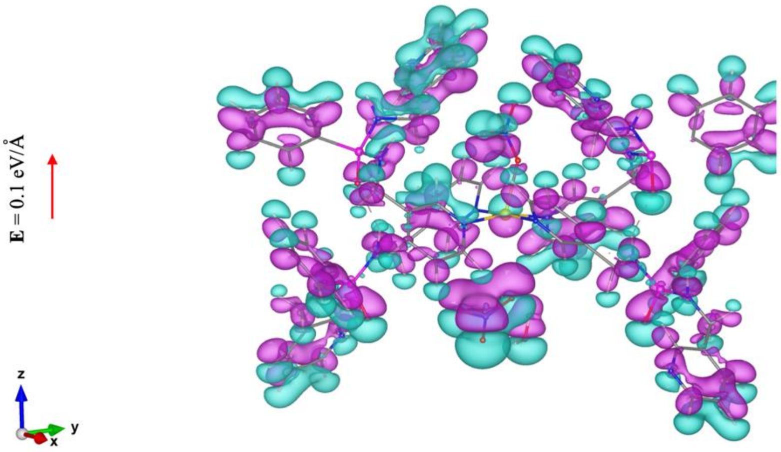

Figure S26: Differential charge density plot under electric field of 0.1 eV/Å, showing both the metal-centered and ligand-centered responses.

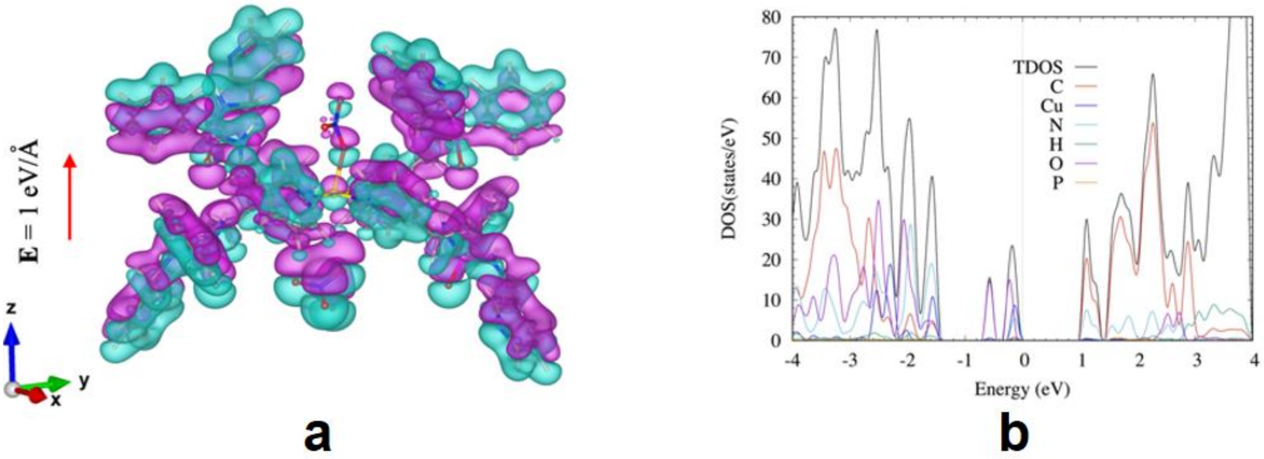

Figure S27: (a) Differential electronic charge density under an electric field applied along the z direction with a magnitude of 1 eV/Å. (b) The electronic density of states (DOS) plot for the molecule in the absence of an electric field reveals a band gap of 1 eV. The valence and conduction bands are primarily contributed by C atoms, with Cu states predominantly located in the valence band around -2 eV.

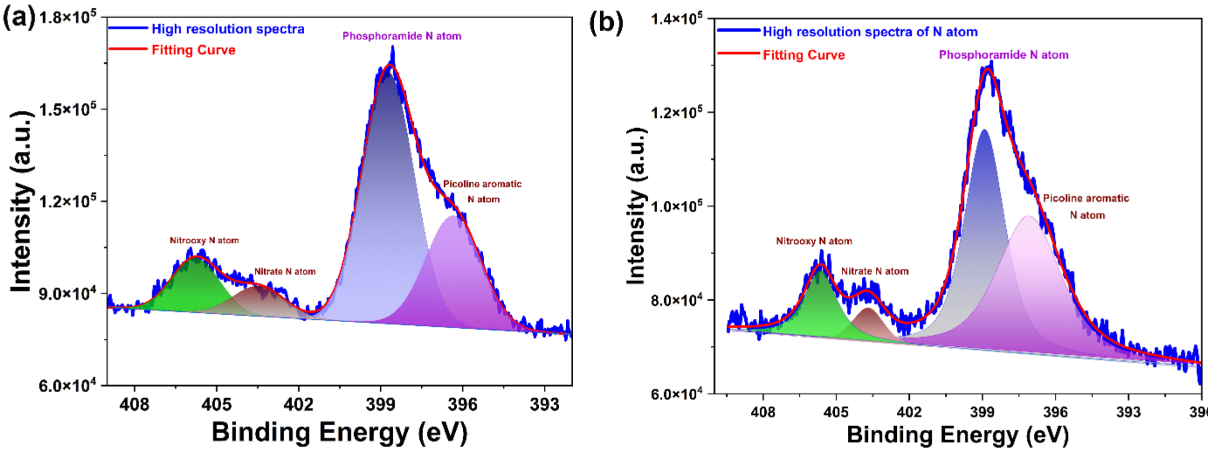

Figure S28: (a) N_{1s} XPS spectra of **1.2H$_2$O** showing the profiles obtained for (a) the unpoled sample and (b) the poled sample.

Table S5: Deconvoluted binding energy of different N_{1s} atoms in poled and unpoled samples.

**Type of nitrogen atom**	**Binding energy of poled sample (eV)**	**Binding energy of unpoled sample (eV)**
Picoline aromatic N_{1s}	397.15	396.37
Phosphoramide N_{1s}	398.97	398.73
Free nitrate N_{1s}	403.69	403.48
Nitrooxy N_{1s}	405.67	405.84

Fabrication and Characterization of 1·2H$_2$O–TPU Composites

Motivated by the intrinsic ferroelectric and piezoelectric properties of compound **1.2H$_2$O**, we sought to harness its functionality for piezoelectric energy harvesting by embedding it in a flexible polymer-based composite film. To prepare the composite film, a piezoelectric-inactive thermoplastic polyurethane (TPU) polymer was used as the substrate. To explore the

mechanical energy harvesting capabilities of compound **1·2H$_2$O**, flexible polymer composites were fabricated by incorporating varying weight percentages (1, 5, 10, 15, and 20 wt%) of powdered crystals of **1·2H$_2$O** into a homogeneous TPU solution in tetrahydrofuran (THF) (Table S6). The resulting slurry was cast onto a PET sheet, air-dried at room temperature, and gently peeled off to yield free-standing composite films (Figure S29). The films exhibited a uniform blue hue and demonstrated excellent mechanical flexibility and durability, retaining structural integrity under repeated folding, rolling, bending, and twisting operations (Figure S30). Powder X-ray diffraction (PXRD) patterns of the composite films revealed well-resolved peaks corresponding to **1·2H$_2$O**, confirming that its crystallinity remained intact within the polymer matrix. Notably, the intensity of diffraction peaks increased progressively with higher filler loading, indicative of enhanced crystallite content at elevated concentrations (Figure S31). From the cross-sectional SEM imaging, the thickness of the composite film was found to be approximately 0.22 mm.

Table S6: Details for the preparation of various mass fractions (wt%) of **1·2H$_2$O**–TPU composite films

Composite (wt%)	1·2H$_2$O (in mg)	TPU (in mg)
**1**	4.03	399
**5**	22.21	422
**10**	44.88	404
**15**	74.10	420
**20**	99.5	398

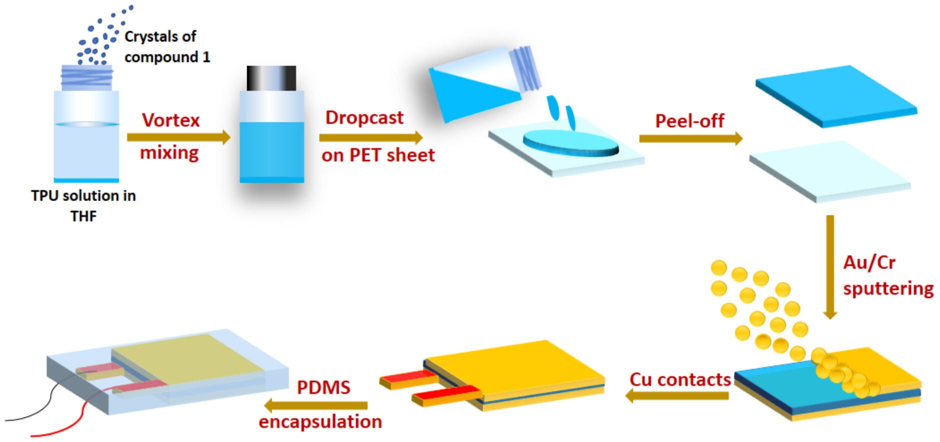

Figure S29: Schematic showing the procedure involved in the preparation of **1·2H$_2$O**–TPU composite films and their fabrication into a device.

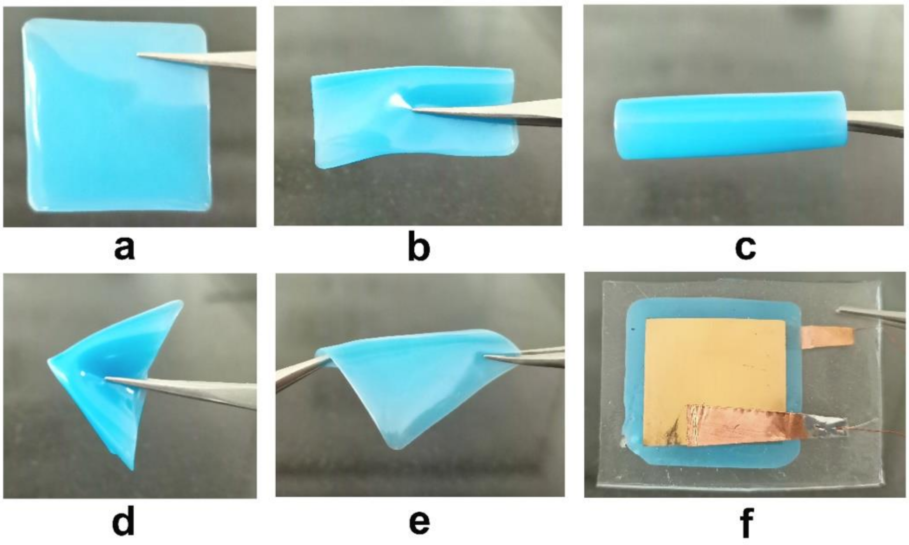

Figure S30: Photographs of (a) the as-made 10 wt% **1·2H$_2$O**–TPU composite film and its flexible nature for (b) folding, (c) rolling, (d) multi-fold bending and (e) twisting operations. (f) Photograph of the as-fabricated **1·2H$_2$O**–TPU composite device.

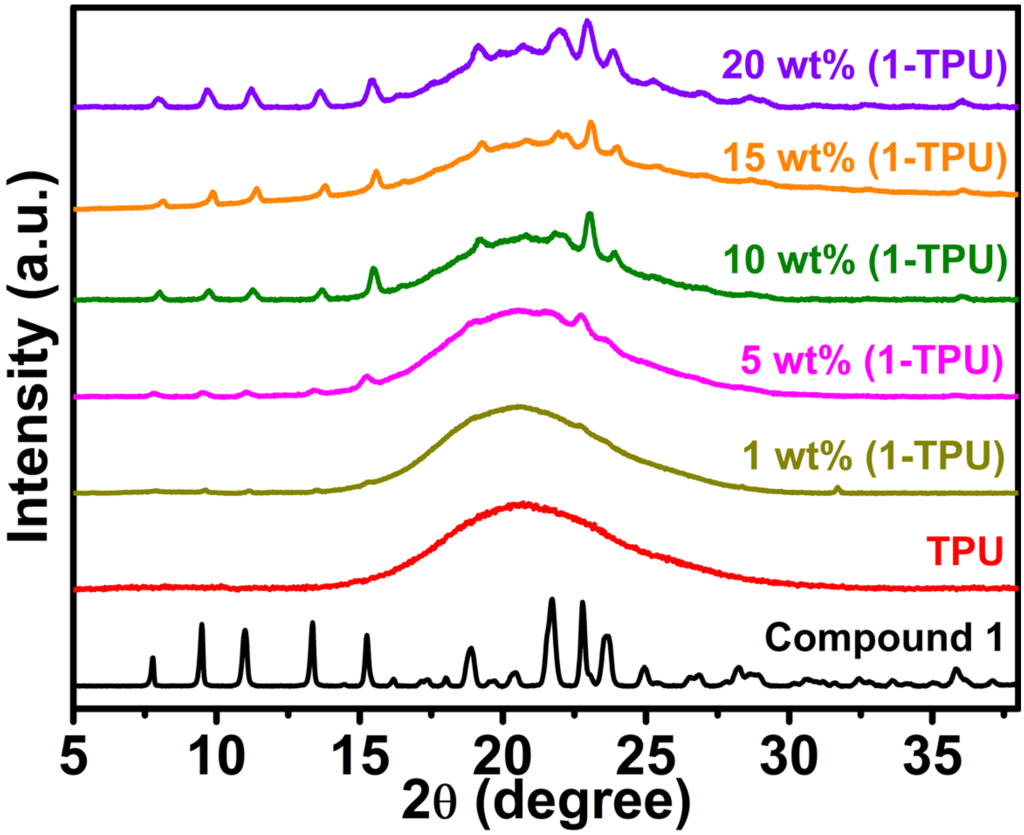

Figure S31: PXRD profiles of composite films of **1·2H$_2$O**–TPU with various wt % of **1·2H$_2$O**.

For device fabrication, a thin bilayer of Au/Cr (100/5 nm) was deposited by magnetron sputtering onto both surfaces of the films to serve as top and bottom electrodes. Copper wires were soldered to copper tape affixed to these electrodes, ensuring reliable electrical

connections. To prevent air-gap formation and to minimize triboelectric interference during dynamic operation, the entire device was encapsulated within a thin, conformal PDMS layer.

All fabricated devices were evaluated under a compressive force of 21 N using a custom-built setup. To maximize energy harvesting efficiency, electrical poling was performed to align the dipoles within the piezoelectric material. Each composite device was subjected to poling under a 6 kV/cm electric field for eight hours, promoting optimal dipole orientation. The energy-harvesting performance of the poled devices was then tested under identical force conditions, with results summarized in Figure S32. Among these composites, the 10 wt% (**1·2H$_2$O**–TPU) device delivered the highest peak-to-peak voltage (V_{PP}) of 25.05 V, identifying it as the optimal concentration for energy-harvesting applications. An asymmetry between positive and negative voltage peaks was observed during pressing and releasing cycles. This phenomenon arises because the pressing response depends primarily on the applied external force, whereas the release is governed by the intrinsic flexibility of the piezocomposite material. The increase in output voltage up to 10 wt% loading is attributed to enhanced interfacial polarization at the interfaces between the hybrid piezoelectric phase and the TPU matrix. However, beyond this threshold, particle aggregation, confirmed by SEM analysis (Figure S33), hinders efficient stress transfer and results in diminished output performance.

Notably, the trend in V_{PP} closely follows the piezoelectric coefficient (d_{33}) values measured by the Berlincourt method (3.4, 16.5, 31.1, 22.7, and 5.3 pC/N for 1, 5, 10, 15, and 20 wt% composites, respectively), further validating the correlation between piezoelectric response and energy-harvesting efficiency (Figure S34). To evaluate the practical performance of the **1·2H$_2$O**–TPU device, output voltage was measured across a range of external resistances from 0.1 to 40 MΩ. The voltage output initially rises with increasing resistance before reaching a plateau, while the current density correspondingly decreases (Figure S35). The device achieved a maximum instantaneous power density of 48.7 μW/cm^2 at an optimal load resistance of 0.9 MΩ. Furthermore, mechanical durability was assessed through repeated compressive and release cycling, during which the device maintained a stable signal amplitude over 1000 cycles, demonstrating its robustness and suitability for real-world ambient energy-harvesting applications (Figure S36). Additionally, the energy generated from the champion 10 wt% **1·2H$_2$O**–TPU device, which exhibited the highest performance, was used to charge commercially available capacitors (10, 47, and 100 μF). The capacitors were connected to the 10 wt% PENG device via a full-wave bridge rectifier, which converted the AC output signals to DC voltages for charging (Figure S37a). The charging rate was higher for smaller capacitors, with the saturation voltage reached within 180 seconds (Figure S37). The stored charge (Q) on each capacitor was calculated using the relation $Q = C_L V$ (where C_L is the capacitance and V is the applied voltage). The stored charge exhibited different trends across capacitors of varying ratings. The 10 μF capacitor stored a relatively small amount of charge, reaching its saturation value rapidly. As the capacitance increased, the storage capacity increased, and the curve became linear for larger capacitors. The 100 μF capacitor stored the maximum charge of 13.44 μC within 450 seconds (Figures S37b and S37c).

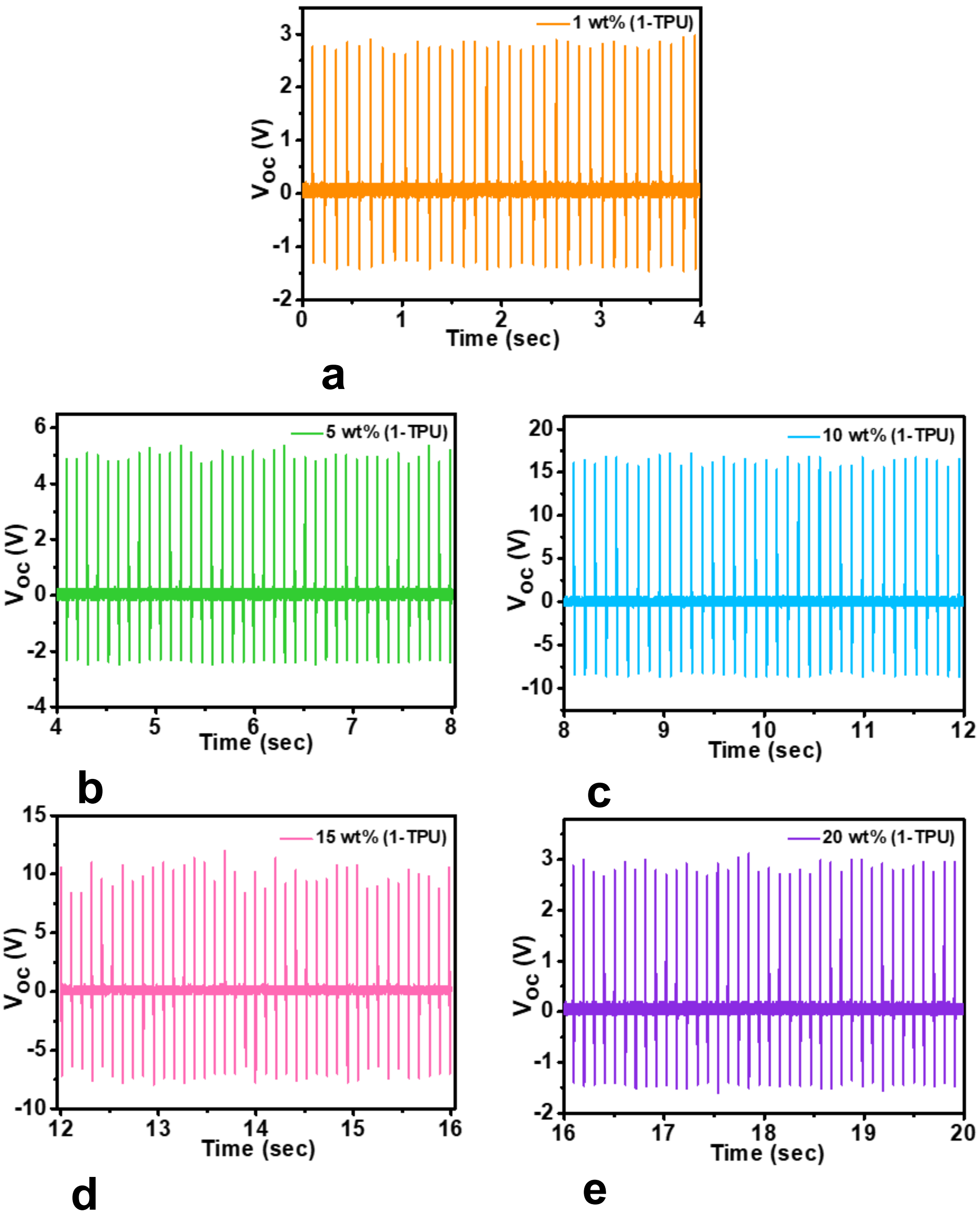

Figure S32: Open-circuit output voltage measurements of various weight percentages (wt%) of poled **1·2H_2O**–TPU composite devices.

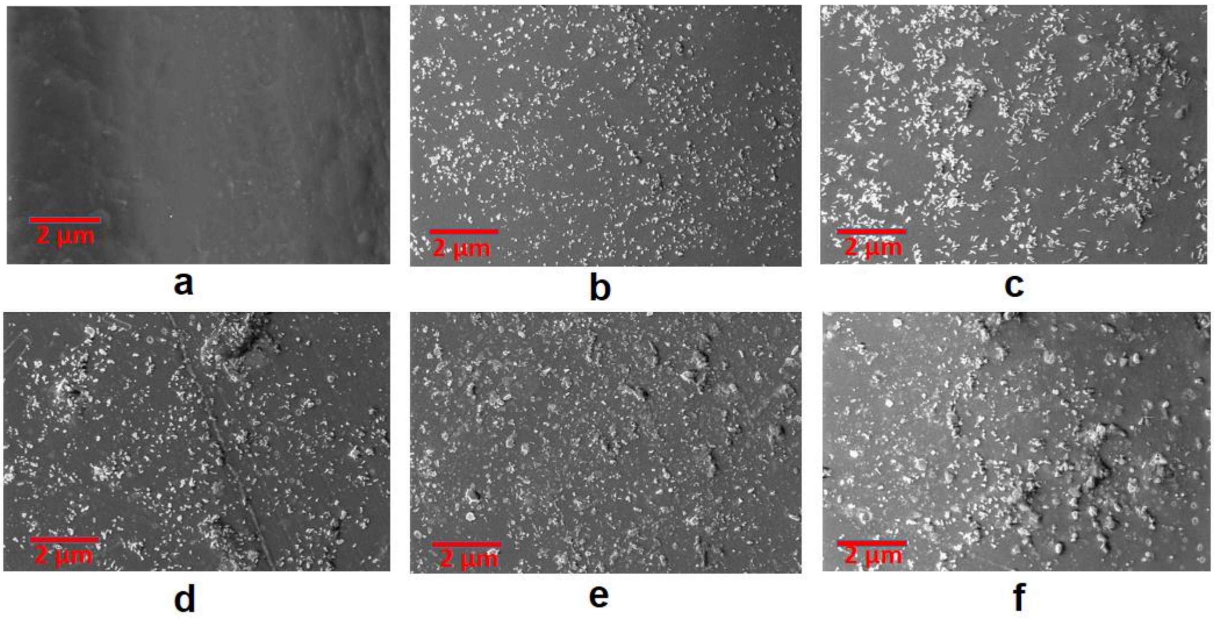

Figure S33: SEM images of (a) neat TPU polymer and (b-f) the respective 1, 5, 10, 15, and 20 wt% of **1·2H_2O**–TPU composite films. The higher wt% composites show significant agglomeration of the crystallites (e-f) compared to the best performing 10 wt% composite (d) resulting in a lower output voltages for the 15 and 20 wt% **1·2H_2O**–TPU devices.

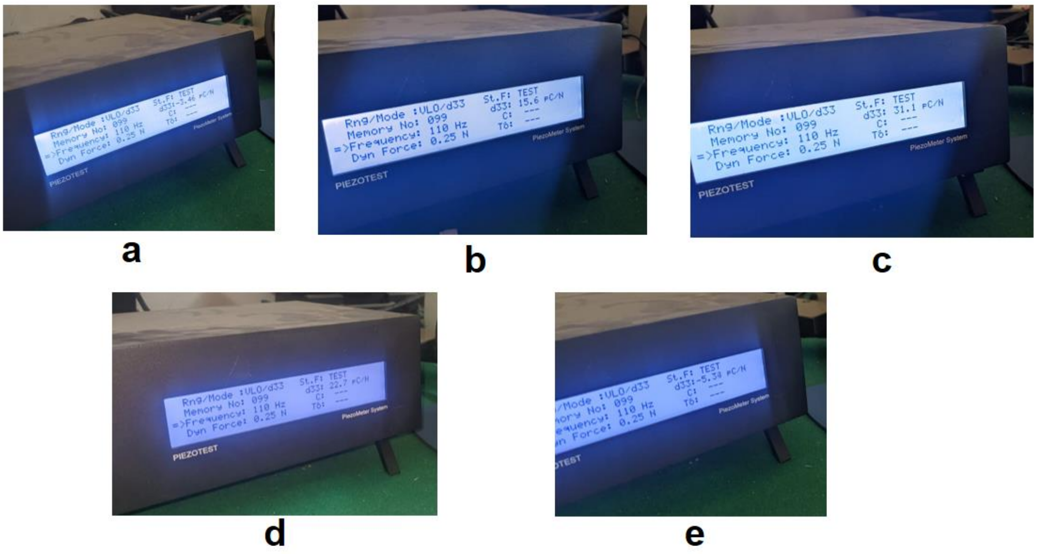

Figure S34: Photographs of the d_{33} meter showing the direct Piezoelectric coefficients (d_{33}) of the poled 1, 5, 10, 15, and 20 wt% of **1·2H_2O**–TPU composite films.

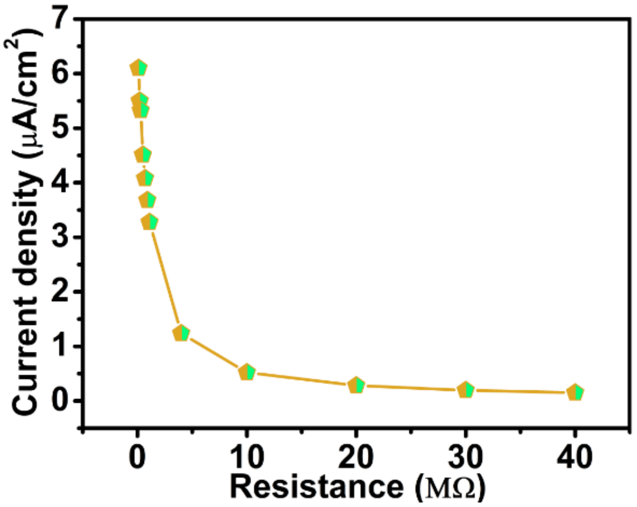

Figure S35: (a) Output voltage and power density of the 10wt% **1·2H_2O**–TPU composite device under different load resistances (solid lines are a guide to the eye). (b) Current density plot of 10wt% **1·2H_2O**–TPU device across various load resistance.

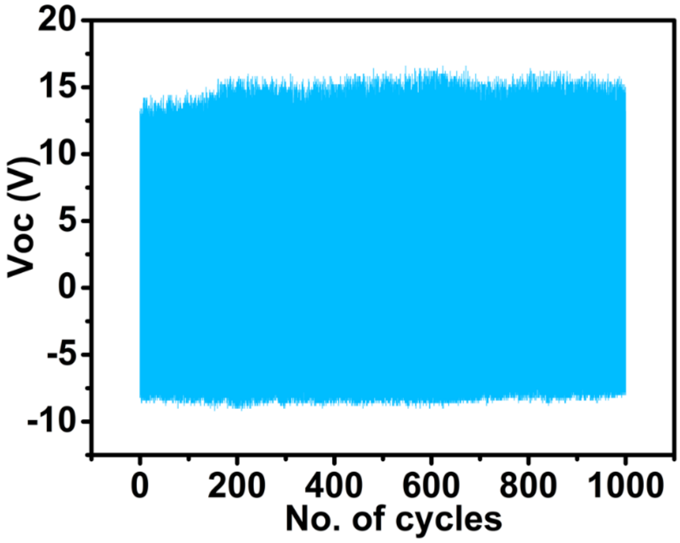

Figure S36: Long-term durability test of the upstanding 10wt% **1·2H_2O**–TPU device with a continuous applied force of 21 N upto 1000 cycles.

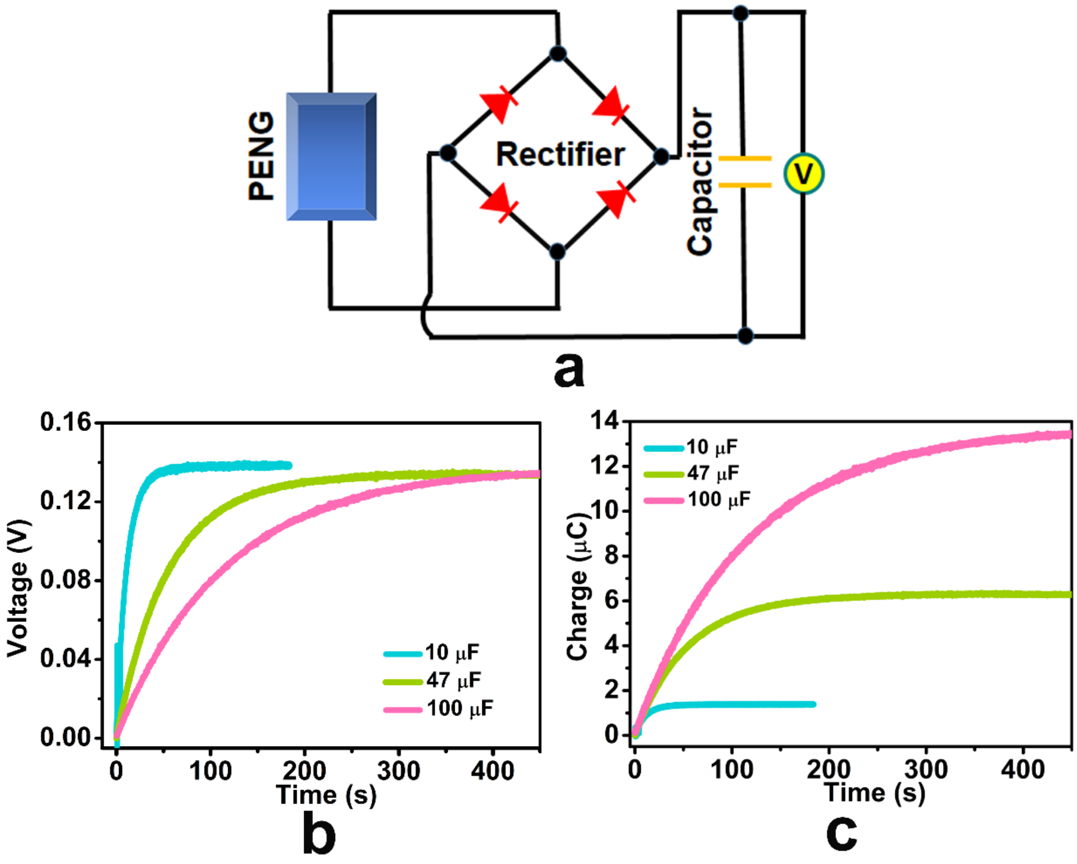

Figure S37: Schematic drawing representation of the charging of the capacitor using a **1**–TPU device connected through a full-wave four diode bridge rectifier circuit. (b) Output saturation voltage of the 10wt% **1·2H$_2$O**–TPU device for different rated capacitances. (c) Plots representing the calculated charge for three different capacitors using output voltages from the 10wt% **1·2H$_2$O**–TPU device.

References

(1) Sheldrick, G. M. Foundations of Crystallography. *Acta Crystallogr. A* **2008**, *64*, 112.
(2) Kurtz, S. K.; Perry, T. T. A Powder Technique for the Evaluation of Nonlinear Optical Materials. *J. Appl. Phys.* **1968**, *39*, 3798.
(3) Graja, A. Production of the Second Harmonic of Light in Ammonium Pentaborate and other Powdered Piezoelectric Crystals. *phys. stat. sol. (b)* **1968**, *27*, K93.
(4) Kohn, W.; Sham, L. J. Self-Consistent Equations Including Exchange and Correlation Effects. *Phys. Rev*. **1965**, *140*, A1133.
(5) Constantin, L. A.; Perdew, J. P.; Pitarke, J. M. Exchange-correlation hole of a generalized gradient approximation for solids and surfaces. *Phys. Rev. B* **2009**, *79*, 075126.
(6) Giannozzi, P.; Baroni, S.; Bonini, N.; Calandra, M.; Car, R.; Cavazzoni, C.; Ceresoli, D.; Chiarotti, G. L.; Cococcioni, M.; Dabo, I.; Dal Corso, A.; de Gironcoli, S.; Fabris, S.; Fratesi, G.; Gebauer, R.; Gerstmann, U.; Gougoussis, C.; Kokalj, A.; Lazzeri, M.; Martin-Samos, L.; Marzari, N.; Mauri, F.; Mazzarello, R.; Paolini, S.; Pasquarello, A.; Paulatto, L.; Sbraccia, C.; Scandolo, S.; Sclauzero, G.; Seitsonen, A. P.; Smogunov, A.; Umari, P.; Wentzcovitch, R. M.

QUANTUM ESPRESSO: a modular and open-source software project for quantum simulations of materials. *J. Phys.: Condens. Matter* **2009**, *21*, 395502.
(7) Kunc, K.; Resta, R. External Fields in the Self-Consistent Theory of Electronic States: A New Method for Direct Evaluation of Macroscopic and Microscopic Dielectric Response. *Phys. Rev. Lett.* **1983**, *51*, 686.
